# Supplementary material for: The Relationship between Metabolically Healthy Obesity and the Risk of Cardiovascular Disease: A Systematic Review and Meta-Analysis
Source: J Clin Med. 2019 Aug 15;8(8):1228. doi: 10.3390/jcm8081228 (PMC6723711; doi:10.3390/jcm8081228)
Supplement: Supplementary file 1 [file jcm-08-01228-s001.pdf]

Supplementary Materials

Appendix S1. PRISMA checklist.

Section/topic	#	Checklist item	Reported on page #
TITLE			
Title	1	Identify the report as a systematic review, meta-analysis, or both.	1
ABSTRACT			
Structured summary	2	Provide a structured summary including, as applicable: background; objectives; data sources; study eligibility criteria, participants, and interventions; study appraisal and synthesis methods; results; limitations; conclusions and implications of key findings; systematic review registration number.	1
INTRODUCTION			
Rationale	3	Describe the rationale for the review in the context of what is already known.	1-2
Objectives	4	Provide an explicit statement of questions being addressed with reference to participants, interventions, comparisons, outcomes, and study design (PICOS).	2
METHODS			
Protocol and registration	5	Indicate if a review protocol exists, if and where it can be accessed (e.g., Web address), and, if available, provide registration information including registration number.	2
Eligibility criteria	6	Specify study characteristics (e.g., PICOS, length of follow-up) and report characteristics (e.g., years considered, language, publication status) used as criteria for eligibility, giving rationale.	2-3
Information sources	7	Describe all information sources (e.g., databases with dates of coverage, contact with study authors to identify additional studies) in the search and date last searched.	2-3
Search	8	Present full electronic search strategy for at least one database, including any limits used, such that it could be repeated.	2-3, Appendix

			S2
Study selection	9	State the process for selecting studies (i.e., screening, eligibility, included in systematic review, and, if applicable, included in the meta-analysis).	3
Data collection process	10	Describe method of data extraction from reports (e.g., piloted forms, independently, in duplicate) and any processes for obtaining and confirming data from investigators.	3
Data items	11	List and define all variables for which data were sought (e.g., PICOS, funding sources) and any assumptions and simplifications made.	3
Risk of bias in individual studies	12	Describe methods used for assessing risk of bias of individual studies (including specification of whether this was done at the study or outcome level), and how this information is to be used in any data synthesis.	3
Summary measures	13	State the principal summary measures (e.g., risk ratio, difference in means).	3
Synthesis of results	14	Describe the methods of handling data and combining results of studies, if done, including measures of consistency (e.g., I^2) for each meta-analysis.	3
Section/topic	#	Checklist item	Reported on page #
Risk of bias across studies	15	Specify any assessment of risk of bias that may affect the cumulative evidence (e.g., publication bias, selective reporting within studies).	3
Additional analyses	16	Describe methods of additional analyses (e.g., sensitivity or subgroup analyses, meta-regression), if done, indicating which were pre-specified.	3
RESULTS			
Study selection	17	Give numbers of studies screened, assessed for eligibility, and included in the review, with reasons for exclusions at each stage, ideally with a flow diagram.	4-5
Study characteristics	18	For each study, present characteristics for which data were extracted (e.g., study size, PICOS, follow-up period) and provide the citations.	4-5, Table S2

Risk of bias within studies	19	Present data on risk of bias of each study and, if available, any outcome level assessment (see item 12).	5-6, Table S1
Results of individual studies	20	For all outcomes considered (benefits or harms), present, for each study: (a) simple summary data for each intervention group (b) effect estimates and confidence intervals, ideally with a forest plot.	5-10
Synthesis of results	21	Present the main results of the review. If meta-analyses are done, include for each, confidence intervals and measures of consistency	5-10
Risk of bias across studies	22	Present results of any assessment of risk of bias across studies (see Item 15).	7, 10, Figure S7, S17
Additional analysis	23	Give results of additional analyses, if done (e.g., sensitivity or subgroup analyses, meta-regression [see Item 16]).	5-10
DISCUSSION			
Summary of evidence	24	Summarize the main findings including the strength of evidence for each main outcome; consider their relevance to key groups (e.g., healthcare providers, users, and policy makers).	10-11
Limitations	25	Discuss limitations at study and outcome level (e.g., risk of bias), and at review-level (e.g., incomplete retrieval of identified research, reporting bias).	11-12
Conclusions	26	Provide a general interpretation of the results in the context of other evidence, and implications for future research.	12
FUNDING			
Funding	27	Describe sources of funding for the systematic review and other support (e.g., supply of data); role of funders for the systematic review.	N/A

Appendix S2. Search strategy.

Main results:

Databases	Articles	Date	Others
PubMed	5101	20190409	
Cochrane CDSR	19	20190409	
Cochrane CENTRAL	716	20190409	including registered clinical trials in clinicaltrials.gov (Started from March, 2019)
EMBASE	5467	20190409	Elsevier platform
CINAHL	788	20190409	
Total	12091		
endnote duplicate	1439		
Total without duplicated	10652		

Keywords:

**PICOS**		**MeSH terms + free text word**	**EMTree terms + free text word**
P	adult		
I	MHO	Obesity, Metabolically Benign OR Metabolically Benign Obesity OR Metabolically Healthy Obesity OR MHO OR obesity paradox OR obesity phenotypes	metabolically benign obesity OR 'obesity paradox'/exp OR obesity paradox OR obesity phenotypes

		<u>Overweight</u> OR <u>obesity</u> OR obese OR <u>BODY MASS INDEX</u> OR BMI OR Quetelet Index OR fat OR <u>Body Weight</u> OR <u>Body Composition</u> OR <u>Anthropometry OR adiposity</u> OR <u>body composition</u> OR body fat OR fatness OR body mass AND*	<u>obesity</u> OR adiposit* OR obesitas OR overweight OR obese OR fat OR <u>body mass</u> OR BMI OR body mass index OR Quetelet index OR <u>Body Weight</u> OR <u>Body Composition</u> OR <u>anthropometry</u> OR anthropometric OR adiposity AND
		Metabolic AND*	metaboli* AND
		normal OR healthy OR benign OR without OR absence	normal OR healthy OR benign OR without OR absence
C	MH-NW	metabolically healthy normal weight OR MHNW	
O	CVD	<u>Cardiovascular Diseases</u> OR cardiovascular OR <u>myocardial infarction</u> OR coronary OR <u>heart failure</u> OR angina pectoris OR stroke OR CVD OR OR <u>Heart Diseases</u> OR Cardiac Disease OR <u>Vascular Diseases</u> OR Cerebrovascular Accident OR cerebrovascular disease OR <u>Carotid Artery Diseases</u>	<u>Cardiovascular Disease</u> OR angiocardio* cardiovascular OR <u>cerebrovascular accident</u>
Study		NOT ((letter[Publication Type]) OR editorial[Publication Type]))	

Search strategy in Pubmed

**PICO**	**Search**	**Query**	**Items found**	**Time**
**PIO**	#55	Search (((((((((Obesity, Metabolically Benign) OR Metabolically Benign Obesity) OR Metabolically Healthy Obesity) OR MHO[Title/Abstract] OR obesity paradox OR obesity phenotypes OR obesity phenotype*) OR (((((((((((((((Overweight) OR obesity) OR obese[Title/Abstract]) OR Body Mass Index) OR BMI[Title/Abstract]) OR Quetelet Index[Title/Abstract]) OR fat[Title/Abstract]) OR Body Weight) OR Body Composition) OR fatness[Title/Abstract]) OR body mass[Title/Abstract]) OR Anthropometry) OR adiposity)) AND ((normal[Text Word] OR healthy[Text Word] OR benign[Text Word] OR without[Text	5101	01:02:13

		Word] OR absence[Text Word]))) AND metabolic))) AND ((((((((((((Carotid Artery Diseases OR Cardiovascular Diseases) OR myocardial infarction) OR coronary[Text Word]) OR heart failure) OR Cardiac Disease) OR angina pectoris[Text Word]) OR Vascular Diseases) OR Cardiovascular[Text Word]) OR CVD[Title/Abstract]) OR heart diseases OR cerebrovascular disease) OR stroke[Text Word]) OR All cause[Title/Abstract])) AND (((((((Mortality) OR mortalit*[Title/Abstract]) OR Morbidity) OR Morbidities[Text Word]) OR Incidence) OR incident[Title/Abstract]) OR death[Title/Abstract])))))		
**O**	#47	Search ((((((Mortality) OR mortalit*[Title/Abstract]) OR Morbidity) OR Morbidities[Text Word]) OR Incidence) OR incident[Title/Abstract]) OR death[Title/Abstract]	3694056	22:48:17
	#46	Search death[Title/Abstract]	649576	22:47:56
	#45	Search incident[Title/Abstract]	72176	22:47:43
	#44	Search Incidence	2685548	22:46:34
	#43	Search Morbidities[Text Word]	21244	22:46:10
	#42	Search Morbidity	2537043	22:45:26
	#41	Search mortalit*[Title/Abstract]	688051	22:45:07
	#40	Search Mortality	1151016	22:44:47
**I**	#39	Search (((((((((((Cardiovascular Diseases) OR myocardial infarction) OR coronary[Text Word]) OR heart failure) OR Cardiac Disease) OR angina pectoris[Text Word]) OR Vascular Diseases) OR Cardiovascular[Text Word]) OR CVD[Title/Abstract]) OR heart diseases) OR stroke[Text Word]) OR All cause[Title/Abstract]	2808604	22:43:03
	#56	Search cerebrovascular disease	364638	01:01:59
	#53	Search Carotid Artery Diseases Sort by: [pubsolr12]	52326	22:56:19
	#38	Search All cause[Title/Abstract]	39382	22:41:54
	#37	Search stroke[Text Word]	262261	22:20:35
	#36	Search heart diseases	1155486	22:20:15
	#35	Search CVD[Title/Abstract]	30938	22:18:54
	#34	Search Cardiovascular[Text Word]	521576	22:18:17

	#33	Search Vascular Diseases	1650271	22:17:14
	#32	Search angina pectoris[Text Word]	41334	22:16:48
	#31	Search Cardiac Disease	1215546	22:16:19
	#30	Search heart failure	234112	22:16:03
	#29	Search coronary[Text Word]	476564	22:15:47
	#28	Search myocardial infarction	237516	22:14:44
	#26	Search Cardiovascular Diseases	2311218	22:13:24
**P**	#25	Search ((((Obesity, Metabolically Benign) OR Metabolically Benign Obesity) OR Metabolically Healthy Obesity) OR MHO[Title/Abstract] OR obesity paradox OR obesity phenotypes OR obesity phenotype*) OR (((((((((((((((Overweight) OR obesity) OR obese[Title/Abstract]) OR Body Mass Index) OR BMI[Title/Abstract]) OR Quetelet Index[Title/Abstract]) OR fat[Title/Abstract]) OR Body Weight) OR Body Composition) OR fatness[Title/Abstract]) OR body mass[Title/Abstract]) OR Anthropometry) OR adiposity)) AND ((normal[Text Word] OR healthy[Text Word] OR benign[Text Word] OR without[Text Word] OR absence[Text Word]))) AND metabolic)	31687	22:12:49
	#24	Search ((((((((((((((Overweight) OR obesity) OR obese[Title/Abstract]) OR Body Mass Index) OR BMI[Title/Abstract]) OR Quetelet Index[Title/Abstract]) OR fat[Title/Abstract]) OR Body Weight) OR Body Composition) OR fatness[Title/Abstract]) OR body mass[Title/Abstract]) OR Anthropometry) OR adiposity)) AND ((normal[Text Word] OR healthy[Text Word] OR benign[Text Word] OR without[Text Word] OR absence[Text Word]))) AND metabolic	30131	22:12:29
	#23	Search metabolic	549733	22:12:19
	#22	Search (normal[Text Word] OR healthy[Text Word] OR benign[Text Word] OR without[Text Word] OR absence[Text Word])	2889081	22:11:57
	#19	Search (((((((((((Overweight) OR obesity) OR obese[Title/Abstract]) OR Body Mass Index) OR BMI[Title/Abstract]) OR Quetelet Index[Title/Abstract]) OR fat[Title/Abstract]) OR Body Weight) OR Body Composition) OR fatness[Title/Abstract]) OR body mass[Title/Abstract]) OR Anthropometry) OR adiposity	1152315	22:08:44

	#18	Search adiposity	26717	22:07:56
	#17	Search Anthropometry	488634	22:07:36
	#16	Search body mass[Title/Abstract]	192274	22:06:08
	#15	Search fatness[Title/Abstract]	3988	22:05:45
	#14	Search Body Composition	83042	22:05:12
	#13	Search Body Weight	603456	22:04:55
	#12	Search fat[Title/Abstract]	239725	22:02:33
	#11	Search Quetelet Index[Title/Abstract]	487	22:02:13
	#10	Search BMI[Title/Abstract]	127098	22:01:56
	#9	Search Body Mass Index	219076	22:01:05
	#8	Search obese[Title/Abstract]	114942	22:00:21
	#7	Search obesity	301266	22:00:02
	#6	Search Overweight	222704	21:59:45
	#5	obesity paradox OR obesity phenotypes OR obesity phenotype*	13269	
	#4	Search MHO[Title/Abstract]	508	21:55:40
	#3	Search Metabolically Healthy Obesity	952	21:54:58
	#2	Search Metabolically Benign Obesity	192	21:54:47
	#1	Search Obesity, Metabolically Benign	192	21:54:33

Search strategy in Embase

PICO	Search	Query	Items found
	#41	#38 AND ('Article'/it OR 'Article in Press'/it OR 'Conference Paper'/it OR 'Conference Review'/it OR 'Review'/it OR 'Short Survey'/it)	5467
	#40	#38 AND 'Conference Abstract'/it	4306
PICO	#39	#20 AND #30 AND #38	9936
O	#38	#30 OR #31 OR #32 OR#33 OR #34 OR #35 OR #36	3043929

	#37	incidence:ti,ab OR incident:ti,ab	1035680
	#36	'incidence'/exp	410146
	#35	morbidities:ti,ab	44807
	#34	'morbidity'/exp	333697
	#33	mortalit*:ti,ab	981914
	#32	'death':ab,ti	891974
	#31	'mortality'/exp	985538
I	#30	#28 OR #29	5118917
	#29	all NEXT/2 cause	68691
	#28	#21 OR #22 OR #23 OR #24 OR #25 OR #26 OR #27	5100231
	#27	'cardiovascular':ab,ti OR 'myocardial':ab,ti OR 'coronary':ab,ti OR 'heart':ab,ti OR 'angina pectoris':ab,ti OR 'stroke':ab,ti OR 'cvd':ab,ti OR 'cardiac':ab,ti OR 'vascular':ab,ti OR 'cerebrovascular':ab,ti	3032451
	#26	'carotid artery disease'/exp	64684
	#25	'cerebrovascular accident'/exp	298467
	#24	'vascular disease'/exp	2505922
	#23	'myocardial disease'/exp	350364
	#22	'heart disease'/exp	1827728
	#21	'cardiovascular disease'/exp	4078463
P	#20	#1 OR #2 OR #3 OR #4 OR #5 OR #6 OR #19	90677
	#19	#6 AND #16 AND #17	87782
	#18	'normal':ti,ab,kw OR 'healthy':ti,ab,kw OR 'benign':ti,ab,kw OR 'without':ti,ab,kw OR 'absence':ti,ab,kw	5632775
	#17	#7 OR #8 OR #9 OR #10 OR #11 OR #12 OR #13 OR #14 OR #15 OR #16	1564413
	#16	'anthropometric':ti,ab,kw OR 'adiposity':ti,ab,kw	84531
	#15	'anthropometry'/exp	78307
	#14	'body composition'/exp	92941
	#13	'body weight'/exp	623402

	#12	'bmi':ti,ab,kw OR 'body mass index':ti,ab,kw OR 'body mass':ti,ab,kw	422618
	#11	'body mass'/exp	367311
	#10	'obesity':ti,ab,kw	335780
	#9	'adiposit*':ti,ab,kw OR 'obesitas':ti,ab,kw OR 'overweight':ti,ab,kw OR 'obese':ti,ab,kw OR 'fat':ti,ab,kw	508795
	#8	'obesity'/exp	471232
	#7	metaboli*:ti,ab	1380617
	#6	'obesity phenotypes' OR (('obesity'/exp OR obesity) AND phenotypes)	6568
	#5	obesity NEAR/3 paradox	1730
	#4	obesity paradox'/exp	28
	#3	mho:ti,ab	794
	#2	'metabolically healthy obesity'/exp	80
	#1	'metabolically benign obesity'/exp	42

Search strategy in Cochrane Library

**PICO**	**Search**	**Query**
	#1	MeSH descriptor: [Obesity, Metabolically Benign] explode all trees
	#2	(Metabolically Healthy Obesity):ti,ab,kw OR (Metabolically Benign Obesity):ti,ab,kw OR (MHO):ti,ab,kw OR (obesity paradox):ti,ab,kw OR obesity paradox OR obesity phenotypes OR obesity phenotype* (Word variations have been searched)
	#3	#1 OR #2
	#4	(Overweight OR obesity OR obese OR Body Mass Index OR BMI OR Quetelet Index OR fat OR Body Weight OR Body Composition OR fatness OR body mass OR Anthropometry OR adiposity):ti,ab,kw (Word variations have been searched)
	#5	(metaboli*):ti,ab,kw (Word variations have been searched)
	#6	(normal OR healthy OR benign OR without OR absence):ti,ab,kw (Word variations have been searched)
	#7	#4 AND #5 AND #6
**P**	#8	#3 OR #7

	#9	MeSH descriptor: [Cardiovascular Diseases] explode all trees
	#10	MeSH descriptor: [Heart Diseases] explode all trees
	#11	MeSH descriptor: [Vascular Diseases] explode all trees
	#12	MeSH descriptor: [Myocardial Infarction] explode all trees
	#13	(cardiovascular OR myocardial OR coronary OR stroke OR CVD):ti,ab,kw (Word variations have been searched)
	#14	(Heart OR Cardiac OR Vascular OR Cerebrovascular):ti,ab,kw (Word variations have been searched)
	#15	(all cause):ti,ab,kw (Word variations have been searched)
**I**	#16	#9 OR #10 OR #11 OR #12 OR #13 OR #14 OR #15
	#17	MeSH descriptor: [Mortality] explode all trees
	#18	MeSH descriptor: [Morbidity] explode all trees
	#19	MeSH descriptor: [Incidence] explode all trees
	#20	(Morbidity OR Morbidities OR Mortality OR mortalities OR Death Rate OR Incidence):ti,ab,kw (Word variations have been searched)
**O**	#21	#17 OR #18 OR #19 OR #20
**PIO**	**#22**	**#8 AND #16 AND #21**

Appendix S3. List of included and excluded studies.

Included studies by hand-made searching

1. Hosseinpanah, F.; Barzin, M.; Sheikholeslami, F.; Azizi, F. Effect of different obesity phenotypes on cardiovascular events in tehran lipid and glucose study (tlgs). *Am. J. Cardiol.* **2011**, *107*, 412-416.
2. Flint, A.J.; Hu, F.B.; Glynn, R.J.; Caspard, H.; Manson, J.E.; Willett, W.C.; Rimm, E.B. Excess weight and the risk of incident coronary heart disease among men and women. *Obesity* **2010**, *18*, 377-383.

Included studies by databases searching

1. Kip, K.E.; Marroquin, O.C.; Kelley, D.E.; Johnson, B.D.; Kelsey, S.F.; Shaw, L.J.; Rogers, W.J.; Reis, S.E. Clinical importance of obesity versus the metabolic syndrome in cardiovascular risk in women: A report from the women's ischemia syndrome evaluation (wise) study. *Circulation* **2004**, *109*, 706-713.
2. Katzmarzyk, P.T.; Church, T.S.; Janssen, I.; Ross, R.; Blair, S.N. Metabolic syndrome, obesity, and mortality: Impact of cardiorespiratory fitness. *Diabetes Care* **2005**, *28*, 391-397.
3. St-Pierre, A.C.; Cantin, B.; Mauriege, P.; Bergeron, J.; Dagenais, G.R.; Despres, J.P.; Lamarche, B. Insulin resistance syndrome, body mass index and the risk of ischemic heart disease. *CMAJ* **2005**, *172*, 1301-1305.
4. Katzmarzyk, P.T.; Janssen, I.; Ross, R.; Church, T.S.; Blair, S.N. The importance of waist circumference in the definition of metabolic syndrome. *Prospective analyses of mortality in men* **2006**, *29*, 404-409.
5. Meigs, J.B.; Wilson, P.W.F.; Fox, C.S.; Vasan, R.S.; Nathan, D.M.; Sullivan, L.M.; D'Agostino, R.B. Body mass index, metabolic syndrome, and risk of type 2 diabetes or cardiovascular disease. *Journal of Clinical Endocrinology and Metabolism* **2006**, *91*, 2906-2912.
6. Song, Y.; Manson, J.E.; Meigs, J.B.; Ridker, P.M.; Buring, J.E.; Liu, S. Comparison of usefulness of body mass index versus metabolic risk factors in predicting 10-year risk of cardiovascular events in women. *American Journal of Cardiology* **2007**, *100*, 1654-1658.
7. Kuk, J.L.; Ardern, C.I. Are metabolically normal but obese individuals at lower risk for all-cause mortality? *Diabetes Care* **2009**, *32*, 2297-2299.
8. Arnlov, J.; Ingelsson, E.; Sundstrom, J.; Lind, L. Impact of body mass index and the metabolic syndrome on the risk of cardiovascular disease and death in middle-aged men. *Circulation* **2010**, *121*, 230-236.
9. Flint, A.J.; Hu, F.B.; Glynn, R.J.; Caspard, H.; Manson, J.E.; Willett, W.C.; Rimm, E.B. Excess weight and the risk of incident coronary heart disease among men and women. *Obesity* **2010**, *18*, 377-383.
10. Hosseinpanah, F.; Barzin, M.; Sheikholeslami, F.; Azizi, F. Effect of different obesity phenotypes on cardiovascular events in tehran lipid and glucose study (tlgs). *Am J Cardiol* **2011**, *107*, 412-416.

11. Voulgari, C.; Tentolouris, N.; Dilaveris, P.; Tousoulis, D.; Katsilambros, N.; Stefanadis, C. Increased heart failure risk in normal-weight people with metabolic syndrome compared with metabolically healthy obese individuals. *Journal of the American College of Cardiology* **2011**, *58*, 1343-1350.
12. Bo, S.; Musso, G.; Gambino, R.; Villois, P.; Gentile, L.; Durazzo, M.; Cavallo-Perin, P.; Cassader, M. Prognostic implications for insulin-sensitive and insulin-resistant normal-weight and obese individuals from a population-based cohort. *Am. J. Clin. Nutr.* **2012**, *96*, 962-969.
13. Durward, C.M.; Hartman, T.J.; Nickols-Richardson, S.M. All-cause mortality risk of metabolically healthy obese individuals in nhanes iii. *Journal of Obesity* **2012**, *2012*.
14. Ogorodnikova, A.D.; Kim, M.; McGinn, A.P.; Muntner, P.; Khan, U.; Wildman, R.P. Incident cardiovascular disease events in metabolically benign obese individuals. *Obesity* **2012**, *20*, 651-659.
15. Appleton, S.L.; Seaborn, C.J.; Visvanathan, R.; Hill, C.L.; Gill, T.K.; Taylor, A.W.; Adams, R.J.; North West Adelaide Health Study, T. Diabetes and cardiovascular disease outcomes in the metabolically healthy obese phenotype: A cohort study. *Diabetes Care* **2013**, *36*, 2388-2394.
16. Choi, K.M.; Cho, H.J.; Choi, H.Y.; Yang, S.J.; Yoo, H.J.; Seo, J.A.; Kim, S.G.; Baik, S.H.; Choi, D.S.; Kim, N.H. Higher mortality in metabolically obese normal-weight people than in metabolically healthy obese subjects in elderly koreans. *Clinical Endocrinology* **2013**, *79*, 364-370.
17. Hanks, L.J.; Tanner, R.M.; Muntner, P.; Kramer, H.; McClellan, W.M.; Warnock, D.G.; Judd, S.E.; Gutiérrez, O.M. Metabolic subtypes and risk of mortality in normal weight, overweight, and obese individuals with ckd. *Clinical Journal of the American Society of Nephrology* **2013**, *8*, 2064-2071.
18. Hinnouho, G.M.; Czernichow, S.; Dugravot, A.; Batty, G.D.; Kivimaki, M.; Singh-Manoux, A. Metabolically healthy obesity and risk of mortality: Does the definition of metabolic health matter? *Diabetes Care* **2013**, *36*, 2294-2300.
19. Ortega, F.B.; Lee, D.C.; Katzmarzyk, P.T.; Ruiz, J.R.; Sui, X.; Church, T.S.; Blair, S.N. The intriguing metabolically healthy but obese phenotype: Cardiovascular prognosis and role of fitness. *European Heart Journal* **2013**, *34*, 389-397.
20. Aung, K.; Lorenzo, C.; Hinojosa, M.A.; Haffner, S.M. Risk of developing diabetes and cardiovascular disease in metabolically unhealthy normal-weight and metabolically healthy obese individuals. *Journal of Clinical Endocrinology and Metabolism* **2014**, *99*, 462-468.
21. Morkedal, B.; Vatten, L.J.; Romundstad, P.R.; Laugsand, L.E.; Janszky, I. Risk of myocardial infarction and heart failure among metabolically healthy but obese individuals: Hunt (nord-trondelag health study), norway. *J Am Coll Cardiol* **2014**, *63*, 1071-1078.
22. Thomsen, M.; Nordestgaard, B.G. Myocardial infarction and ischemic heart disease in overweight and obesity with and without metabolic syndrome. *JAMA Internal Medicine* **2014**, *174*, 15-22.
23. van der, A.D.; Nooyens, A.C.; van Duijnhoven, F.J.; Verschuren, M.M.; Boer, J.M. All-cause mortality risk of metabolically healthy abdominal obese individuals: The epic-morgen study. *Obesity (Silver Spring)* **2014**, *22*, 557-564.
24. Hinnouho, G.M.; Czernichow, S.; Dugravot, A.; Nabi, H.; Brunner, E.J.; Kivimaki, M.; Singh-Manoux, A. Metabolically healthy obesity and the risk of

cardiovascular disease and type 2 diabetes: The whitehall ii cohort study. *European Heart Journal* **2015**, *36*, 551-559.
25. Mirbolouk, M.; Asgari, S.; Sheikholeslami, F.; Mirbolouk, F.; Azizi, F.; Hadaegh, F. Different obesity phenotypes, and incident cardiovascular disease and mortality events in elderly iranians: Tehran lipid and glucose study. *Geriatrics and Gerontology International* **2015**, *15*, 449-456.
26. Twig, G.; Gerstein, H.C.; Shor, D.B.A.; Derazne, E.; Tzur, D.; Afek, A.; Tirosh, A. Coronary artery disease risk among obese metabolically healthy young men. *European Journal of Endocrinology* **2015**, *173*, 305-312.
27. Cheng, F.W.; Gao, X.; Mitchell, D.C.; Wood, C.; Rolston, D.D.; Still, C.D.; Jensen, G.L. Metabolic health status and the obesity paradox in older adults. *J. Nutr. Gerontol. Geriatr.* **2016**, *35*, 161-176.
28. Dhana, K.; Koolhaas, C.M.; Van Rossum, E.F.C.; Ikram, M.A.; Hofman, A.; Kavousi, M.; Franco, O.H. Metabolically healthy obesity and the risk of cardiovascular disease in the elderly population. *PLoS ONE* **2016**, *11*.
29. Guo, F.; Garvey, W.T. Cardiometabolic disease risk in metabolically healthy and unhealthy obesity: Stability of metabolic health status in adults. *Obesity* **2016**, *24*, 516-525.
30. Caleyachetty, R.; Thomas, G.N.; Toulis, K.A.; Mohammed, N.; Gokhale, K.M.; Balachandran, K.; Nirantharakumar, K. Metabolically healthy obese and incident cardiovascular disease events among 3.5 million men and women. *Journal of the American College of Cardiology* **2017**, *70*, 1429-1437.
31. Doustmohamadian, S.; Serahati, S.; Barzin, M.; Keihani, S.; Azizi, F.; Hosseinpanah, F. Risk of all-cause mortality in abdominal obesity phenotypes: Tehran lipid and glucose study. *Nutrition, Metabolism and Cardiovascular Diseases* **2017**, *27*, 241-248.
32. Hansen, L.; Netterstrøm, M.K.; Johansen, N.B.; Rønn, P.F.; Vistisen, D.; Husemoen, L.L.N.; Jørgensen, M.E.; Rod, N.H.; Færch, K. Metabolically healthy obesity and ischemic heart disease: A 10-year follow-up of the inter99 study. *Journal of Clinical Endocrinology and Metabolism* **2017**, *102*, 1934-1942.
33. Loprinzi, P.D.; Frith, E. Cardiometabolic healthy obesity paradigm and all-cause mortality risk. *European Journal of Internal Medicine* **2017**, *43*, 42-45.
34. Mirzaei, B.; Abdi, H.; Serahati, S.; Barzin, M.; Niroomand, M.; Azizi, F.; Hosseinpanah, F. Cardiovascular risk in different obesity phenotypes over a decade follow-up: Tehran lipid and glucose study. *Atherosclerosis* **2017**, *258*, 65-71.
35. Al-khalidi, B.; Kimball, S.M.; Kuk, J.L.; Ardern, C.I. Metabolically healthy obesity, vitamin d, and all-cause and cardiometabolic mortality risk in nhanes iii. *Clinical Nutrition* **2018**.
36. Eckel, N.; Li, Y.; Kuxhaus, O.; Stefan, N.; Hu, F.B.; Schulze, M.B. Transition from metabolic healthy to unhealthy phenotypes and association with cardiovascular disease risk across bmi categories in 90 257 women (the nurses' health study): 30 year follow-up from a prospective cohort study. *The Lancet Diabetes and Endocrinology* **2018**, *6*, 714-724.
37. Kuk, J.L.; Rotondi, M.; Sui, X.; Blair, S.N.; Ardern, C.I. Individuals with obesity but no other metabolic risk factors are not at significantly elevated all-cause mortality risk in men and women. *Clinical Obesity* **2018**, *8*, 305-312.

38. Lassale, C.; Tzoulaki, I.; Moons, K.G.M.; Sweeting, M.; Boer, J.; Johnson, L.; Huerta, J.M.; Agnoli, C.; Freisling, H.; Weiderpass, E.*, et al.* Separate and combined associations of obesity and metabolic health with coronary heart disease: A pan-european case-cohort analysis. *European Heart Journal* **2018**, *39*, 397-406.
39. Lee, S.H.; Jeong, M.H.; Kim, J.H.; Kim, M.C.; Sim, D.S.; Hong, Y.J.; Ahn, Y.; Chae, S.C.; Seong, I.W.; Park, J.S.*, et al.* Influence of obesity and metabolic syndrome on clinical outcomes of st-segment elevation myocardial infarction in men undergoing primary percutaneous coronary intervention. *Journal of Cardiology* **2018**, *72*, 328-334.
40. Li, L.; Chen, K.; Wang, A.P.; Gao, J.Q.; Zhao, K.; Wang, H.B.; Dou, J.T.; Lv, Z.H.; Wang, B.A.; Yan, W.H.*, et al.* Cardiovascular disease outcomes in metabolically healthy obesity in communities of beijing cohort study. *International Journal of Clinical Practice* **2018**.
41. Xu, Y.; Li, H.; Wang, A.; Su, Z.; Yang, G.; Luo, Y.; Tao, L.; Chen, S.; Wu, S.; Wang, Y.*, et al.* Association between the metabolically healthy obese phenotype and the risk of myocardial infarction: Results from the kailuan study. *Eur J Endocrinol* **2018**, *179*, 343-352.
42. Zhang, R.; Dong, S.Y.; Wang, W.M.; Fei, S.Y.; Xiang, H.; Zeng, Q. Obesity, metabolic abnormalities, and mortality in older men. *Journal of Geriatric Cardiology* **2018**, *15*, 422-427.
43. Rose, G. Incubation period of coronary heart disease. *Br. Med. J. (Clin. Res. Ed)* **1982**, *284*, 1600-1601.

Excluded studies with reasons (Cross-sectional studies)

1. Chen, X.; Gui, G.; Ji, W.; Xue, Q.; Wang, C.; Li, H. The relationship between obesity subtypes based on bmi and cardio-cerebrovascular disease. *Hypertens. Res.* **2019**.
2. Khawaja, K.I.; Mian, S.A.; Fatima, A.; Tahir, G.M.; Khan, F.F.; Burney, S.; Hasan, A.; Masud, F. Phenotypic and metabolic dichotomy in obesity: Clinical, biochemical and immunological correlates of metabolically divergent obese phenotypes in healthy south asian adults. *Singapore Med. J.* **2018**, *59*, 431-438.
3. Kwon, B.J.; Kim, D.W.; Her, S.H.; Kim, D.B.; Jang, S.W.; Cho, E.J.; Ihm, S.H.; Kim, H.Y.; Youn, H.J.; Seung, K.B.*, et al.* Metabolically obese status with normal weight is associated with both the prevalence and severity of angiographic coronary artery disease. *Metabolism* **2013**, *62*, 952-960.
4. Blake, K.B.; Shankar, A.; Madhavan, S.; Ducatman, A. Associations among cardiometabolic risk factor clustering, weight status, and cardiovascular disease in an appalachian population. *J. Clin. Hypertens. (Greenwich)* **2010**, *12*, 964-972.

Excluded studies with reasons (focused on metabolically healthy overweight / metabolically healthy obesity)

1. Andersen, S.S.; Andersson, C.; Berger, S.M.; Jensen, T.B.; Torp-Pedersen, C.T.; Gislason, G.H.; Køber, L.; Schmiegelow, M.D. Impact of metabolic disorders

on the relation between overweight/obesity and incident myocardial infarction and ischaemic stroke in fertile women: A nationwide cohort study. *Clinical obesity* **2015**, *5*, 127-135.

Excluded studies with reasons (Not relevant to metabolically healthy obesity)

1. Horwich, T.B.; Fonarow, G.C.; Clark, A.L. Obesity and the obesity paradox in heart failure. *Prog. Cardiovasc. Dis.* **2018**, *61*, 151-156.
2. Bolоukat, R.R.; Ramezankhani, A.; Hasheminia, M.; Tasdighi, E.; Azizi, F.; Hadaegh, F. Impact of blood pressure, cholesterol and glucose in the association between adiposity measures and coronary heart disease and stroke among iranian population. *Clin. Nutr.* **2018**, *37*, 2060-2067.
3. Kazempour-Ardebili, S.; Ramezankhani, A.; Eslami, A.; Akbarpour, S.; Azizi, F.; Hadaegh, F. Metabolic mediators of the impact of general and central adiposity measures on cardiovascular disease and mortality risks in older adults: Tehran lipid and glucose study. *Geriatrics and Gerontology International* **2017**, *17*, 2017-2024.
4. Hruby, S.A.; Bulathsinhala, L.; McKinnon, C.J.; Hill, O.T.; Montain, S.J.; Young, A.J.; Smith, T.J. Body mass index at accession and incident cardiometabolic risk factors in us army soldiers, 2001-2011. *PLoS One* **2017**, *12*.
5. Sung, K.C.; Ryu, S.; Lee, J.Y.; Lee, S.; Cheong, E.; Kim, J.Y.; Wild, S.H.; Byrne, C.D. All cause mortality and body mass index in a young asian occupational cohort without baseline metabolic syndrome components. *Int. J. Cardiol.* **2016**, *224*, 271-278.
6. Patsa, C.; Toutouzas, K.; Tsiamis, E.; Tsioufis, C.; Spanos, A.; Karanasos, A.; Michelongona, A.; Tousoulis, D.; Stefanadis, C. Impact of metabolic syndrome on clinical outcomes after new generation drug-eluting stent implantation: The 'obesity paradox' phenomenon is still apparent. *Nutrition, Metabolism and Cardiovascular Diseases* **2013**, *23*, 307-313.
7. Suadicani, P.; Ole Hein, H.; Von Eyben, F.E.; Gyntelberg, F. Metabolic and lifestyle predictors of ischemic heart disease and all-cause mortality among normal weight, overweight, and obese men: A 16-year follow-up in the copenhagen male study. *Metab. Syndr. Relat. Disord.* **2009**, *7*, 97-104.
8. Wienbergen, H.; Gitt, A.K.; Juenger, C.; Schiele, R.; Heer, T.; Towae, F.; Gohlke, H.; Senges, J. Impact of the body mass index on occurrence and outcome of acute st-elevation myocardial infarction. *Clin. Res. Cardiol.* **2008**, *97*, 83-88.
9. Conley, R.R.; Shim, J.C.; Kelly, D.L.; Feldman, S.; Yu, Y.; McMahon, R.P. Cardiovascular disease in relation to weight in deceased persons with schizophrenia. *Compr. Psychiatry* **2005**, *46*, 460-467.
10. Wessel, T.R.; Arant, C.B.; Olson, M.B.; Johnson, B.D.; Reis, S.E.; Sharaf, B.L.; Shaw, L.J.; Handberg, E.; Sopko, G.; Kelsey, S.F.*, et al.* Relationship of physical fitness vs body mass index with coronary artery disease and cardiovascular events in women. *J. Am. Med. Assoc.* **2004**, *292*, 1179-1187.

Excluded studies with reasons (outcomes not interested of our study)

1. Feng, T.; Vegard, M.; Strand, L.B.; Laugsand, L.E.; Mørkedal, B.; Aune, D.; Vatten, L.; Ellekjær, H.; Loennechen, J.P.; Mukamal, K.*, et al.* Metabolically healthy obesity and risk for atrial fibrillation: The hunt study. *Obesity* **2019**, *27*, 332-338.
2. Estrella, M.L.; Pirzada, A.; Durazo-Arvizu, R.A.; Cai, J.; Giachello, A.L.; Espinoza Gacinto, R.; Siega-Riz, A.M.; Daviglus, M.L. Correlates of and body composition measures associated with metabolically healthy obesity phenotype in hispanic/latino women and men: The hispanic community health study/study of latinos (hchs/sol). *J. Obes.* **2019**, *2019*, 1251456.
3. Kowall, B.; Lehmann, N.; Mahabadi, A.A.; Moebus, S.; Erbel, R.; Jockel, K.H.; Stang, A. Associations of metabolically healthy obesity with prevalence and progression of coronary artery calcification: Results from the heinz nixdorf recall cohort study. *Nutr. Metab. Cardiovasc. Dis.* **2018**.
4. Gilardini, L.; Zambon, A.; Soranna, D.; Croci, M.; Invitti, C. Predictors of the transition from metabolically healthy obesity to unhealthy obesity. *Eating and Weight Disorders* **2018**, *23*, 739-744.
5. Chau, K.; Girerd, N.; Magnusson, M.; Lamiral, Z.; Bozec, E.; Merckle, L.; Leosdottir, M.; Bachus, E.; Frikha, Z.; Ferreira, J.P.*, et al.* Obesity and metabolic features associated with long-term developing diastolic dysfunction in an initially healthy population-based cohort. *Clin. Res. Cardiol.* **2018**, *107*, 887-896.
6. Bradshaw, P.T.; Reynolds, K.R.; Wagenknecht, L.E.; Ndumele, C.E.; Stevens, J. Incidence of components of metabolic syndrome in the metabolically healthy obese over 9 years follow-up: The atherosclerosis risk in communities study. *Int. J. Obes.* **2018**, *42*, 295-301.
7. Akinyemiju, T.; Moore, J.X.; Pisu, M.; Judd, S.E.; Goodman, M.; Shikany, J.M.; Howard, V.J.; Safford, M.; Gilchrist, S.C. A prospective study of obesity, metabolic health, and cancer mortality. *Obesity (19307381)* **2018**, *26*, 193-201.
8. Ahmad, M.I.; Li, Y.; Soliman, E.Z. Association of obesity phenotypes with electrocardiographic left ventricular hypertrophy in the general population. *J. Electrocardiol.* **2018**, *51*, 1125-1130.
9. Yoon, J.W.; Jung, C.H.; Kim, M.K.; Park, H.E.; Park, K.S.; Jang, H.C.; Moon, M.K.; Choi, S.Y.; Koo, B.K. Influence of the definition of "metabolically healthy obesity" on the progression of coronary artery calcification. *PLoS One* **2017**, *12*, e0178741.
10. Lee, H.; Choi, E.K.; Lee, S.H.; Han, K.D.; Rhee, T.M.; Park, C.S.; Lee, S.R.; Choe, W.S.; Lim, W.H.; Kang, S.H.*, et al.* Atrial fibrillation risk in metabolically healthy obesity: A nationwide population-based study. *Int. J. Cardiol.* **2017**, *240*, 221-227.
11. Kim, T.J.; Shin, H.Y.; Chang, Y.; Kang, M.; Jee, J.; Choi, Y.H.; Ahn, H.S.; Ahn, S.H.; Son, H.J.; Ryu, S. Metabolically healthy obesity and the risk for subclinical atherosclerosis. *Atherosclerosis* **2017**, *262*, 191-197.
12. Jae, S.Y.; Babu, A.S.; Yoon, E.S.; Kurl, S.; Laukkanen, J.A.; Choi, Y.H.; Franklin, B.A. Impact of cardiorespiratory fitness and risk of systemic hypertension in nonobese versus obese men who are metabolically healthy or unhealthy. *Am. J. Cardiol.* **2017**, *120*, 765-768.
13. De Lorenzo, A.; Glerian, L.; Amaral, A.C.; Reis, T.B.; Lima, R.S.L. "Metabolically healthy" obesity: Prevalence, clinical features and association with

myocardial ischaemia. *Obes. Res. Clin. Pract.* **2017**, *11*, 315-323.
14. Buscemi, S.; Chiarello, P.; Buscemi, C.; Corleo, D.; Massenti, M.F.; Barile, A.M.; Rosafio, G.; Maniaci, V.; Settipani, V.; Cosentino, L.*, et al.* Characterization of metabolically healthy obese people and metabolically unhealthy normal-weight people in a general population cohort of the abcd study. *Journal of diabetes research* **2017**, *2017*, 9294038.
15. Brant, L.C.; Wang, N.; Ojeda, F.M.; LaValley, M.; Barreto, S.M.; Benjamin, E.J.; Mitchell, G.F.; Vasan, R.S.; Palmisano, J.N.; Munzel, T.*, et al.* Relations of metabolically healthy and unhealthy obesity to digital vascular function in three community-based cohorts: A meta-analysis. *Journal of the American Heart Association* **2017**, *6*.
16. Loenneke, J.P.; Loprinzi, P.D. Obesity is associated with insulin resistance but not skeletal muscle dysfunction or all-cause mortality. *Age* **2016**, *38*, 1-7.
17. Kang, Y.M.; Jung, C.H.; Jang, J.E.; Hwang, J.Y.; Kim, E.H.; Park, J.Y.; Kim, H.K.; Lee, W.J. The association of incident hypertension with metabolic health and obesity status: Definition of metabolic health does not matter. *Clin. Endocrinol. (Oxf.)* **2016**, *85*, 207-215.
18. Dobson, R.; Burgess, M.I.; Sprung, V.S.; Irwin, A.; Hamer, M.; Jones, J.; Daousi, C.; Adams, V.; Kemp, G.J.; Shojaee-Moradie, F.*, et al.* Metabolically healthy and unhealthy obesity: Differential effects on myocardial function according to metabolic syndrome, rather than obesity. *Int. J. Obes.* **2016**, *40*, 153-161.
19. Nyström, P.K.; Carlsson, A.C.; Leander, K.; De Faire, U.; Hellenius, M.L.; Gigante, B. Obesity, metabolic syndrome and risk of atrial fibrillation: A swedish, prospective cohort study. *PLoS One* **2015**, *10*.
20. Kim, S.; Kyung, C.; Park, J.S.; Lee, S.P.; Kim, H.K.; Ahn, C.W.; Kim, K.R.; Kang, S. Normal-weight obesity is associated with increased risk of subclinical atherosclerosis. *Cardiovasc. Diabetol.* **2015**, *14*.
21. Hashimoto, Y.; Tanaka, M.; Okada, H.; Senmaru, T.; Hamaguchi, M.; Asano, M.; Yamazaki, M.; Oda, Y.; Hasegawa, G.; Toda, H.*, et al.* Metabolically healthy obesity and risk of incident ckd. *Clin. J. Am. Soc. Nephrol.* **2015**, *10*, 578-583.
22. Eckel, N.; Mühlenbruch, K.; Meidtner, K.; Boeing, H.; Stefan, N.; Schulze, M.B. Characterization of metabolically unhealthy normal-weight individuals: Risk factors and their associations with type 2 diabetes. *Metabolism* **2015**, *64*, 862-871.
23. Benziger, C.P.; Bernabé-Ortiz, A.; Gilman, R.H.; Checkley, W.; Smeeth, L.; Málaga, G.; Miranda, J.J.; Casas, J.P.; Smith, G.D.; Ebrahim, S.*, et al.* Metabolic abnormalities are common among south american hispanics subjects with normal weight or excess body weight: The cronicas cohort study. *PLoS One* **2015**, *10*.
24. Benckert, M.; Lilja, M.; Söderberg, S.; Eliasson, M. Improved metabolic health among the obese in six population surveys 1986 to 2009: The northern sweden monica study. *BMC obesity* **2015**, *2*.
25. Achilike, I.; Hazuda, H.P.; Fowler, S.P.; Aung, K.; Lorenzo, C. Predicting the development of the metabolically healthy obese phenotype. *Int. J. Obes.*

2015, *39*, 228-234.
26. Hosseinpanah, F.; Barzin, M.; Amiri, P.; Azizi, F. The trends of metabolic syndrome in normal-weight tehranian adults. *Ann. Nutr. Metab.* **2011**, *58*, 126-132.

Excluded studies with reasons (Not comparing metabolically healthy obesity with metabolically healthy normal weight)

1. Park, Y.M.M.; Choi, M.K.; Lee, S.S.; Shivappa, N.; Han, K.; Steck, S.E.; Hébert, J.R.; Merchant, A.T.; Sandler, D.P. Dietary inflammatory potential and risk of mortality in metabolically healthy and unhealthy phenotypes among overweight and obese adults. *Clin. Nutr.* **2019**, *38*, 682-688.
2. Li, H.; He, D.; Zheng, D.; Amsalu, E.; Wang, A.; Tao, L.; Guo, J.; Li, X.; Wang, W.; Guo, X. Metabolically healthy obese phenotype and risk of cardiovascular disease: Results from the china health and retirement longitudinal study. *Arch. Gerontol. Geriatr.* **2019**, *82*, 1-7.
3. Li, H.; He, D.; Zheng, D.; Amsalu, E.; Wang, A.; Tao, L.; Guo, J.; Li, X.; Wang, W.; Guo, X. Metabolically healthy obese phenotype and risk of cardiovascular disease: Results from the china health and retirement longitudinal study. *Arch. Gerontol. Geriatr.* **2019**, *82*, 1-7.
4. Kouvari, M.; Panagiotakos, D.B.; Yannakoulia, M.; Georgousopoulou, E.; Critselis, E.; Chrysohoou, C.; Tousoulis, D.; Pitsavos, C. Transition from metabolically benign to metabolically unhealthy obesity and 10-year cardiovascular disease incidence: The attica cohort study. *Metabolism* **2019**, *93*, 18-24.
5. Echouffo-Tcheugui, J.B.; Short, M.I.; Xanthakis, V.; Field, P.; Sponholtz, T.R.; Larson, M.G.; Vasan, R.S. Natural history of obesity subphenotypes: Dynamic changes over two decades and prognosis in the framingham heart study. *The Journal of clinical endocrinology and metabolism* **2019**, *104*, 738-752.
6. Cho, Y.K.; Kang, Y.M.; Yoo, J.H.; Lee, J.; Park, J.Y.; Lee, W.J.; Kim, Y.J.; Jung, C.H. Implications of the dynamic nature of metabolic health status and obesity on risk of incident cardiovascular events and mortality: A nationwide population-based cohort study. *Metabolism* **2019**.
7. Mongraw-Chaffin, M.; Foster, M.C.; Anderson, C.A.M.; Burke, G.L.; Haq, N.; Kalyani, R.R.; Ouyang, P.; Sibley, C.T.; Tracy, R.; Woodward, M.*, et al.* Metabolically healthy obesity, transition to metabolic syndrome, and cardiovascular risk. *J. Am. Coll. Cardiol.* **2018**, *71*, 1857-1865.
8. Lee, H.J.; Choi, E.K.; Lee, S.H.; Kim, Y.J.; Han, K.D.; Oh, S. Risk of ischemic stroke in metabolically healthy obesity: A nationwide population-based study. *PLoS One* **2018**, *13*, e0195210.
9. Johnson, W.; Bell, J.A.; Robson, E.; Norris, T.; Kivimäki, M.; Hamer, M. Do worse baseline risk factors explain the association of healthy obesity with increased mortality risk? Whitehall ii study. *Int. J. Obes.* **2018**.
10. Jae, S.Y.; Kurl, S.; Fernhall, B.; Kunutsor, S.K.; Franklin, B.A.; Laukkanen, J.A. Are metabolically healthy overweight/obese men at increased risk of sudden cardiac death? *Mayo Clin. Proc.* **2018**, *93*, 1266-1270.
11. Espinosa De Ycaza, A.E.; Donegan, D.; Jensen, M.D. Long-term metabolic risk for the metabolically healthy overweight/obese phenotype. *Int. J. Obes.*

2018, *42*, 302-309.
12. Echouffo-Tcheugui, J.B.; Short, M.I.; Xanthakis, V.; Field, P.; Sponholtz, T.R.; Larson, M.G.; Vasan, R.S. Natural history of obesity subphenotypes: Dynamic changes over two decades and prognosis in the framingham heart study. *J. Clin. Endocrinol. Metab.* **2018**, N.PAG-N.PAG.
13. De Lorenzo, A.; Carazza, M.; Lima, R. All-cause mortality of metabolically healthy or unhealthy obese: Risk stratification using myocardial perfusion imaging. *Archives of medical sciences. Atherosclerotic diseases* **2018**, *3*, e90-e95.
14. Sánchez-Iñigo, L.; Navarro-González, D.; Fernández-Montero, A.; Pastrana-Delgado, J.; Martínez, J.A. Risk of incident ischemic stroke according to the metabolic health and obesity states in the vascular-metabolic cun cohort. *Int. J. Stroke* **2017**, *12*, 187-191.
15. Hulten, E.A.; Bittencourt, M.S.; Preston, R.; Singh, A.; Romagnolli, C.; Ghoshhajra, B.; Shah, R.; Abbasi, S.; Abbara, S.; Nasir, K.*, et al.* Obesity, metabolic syndrome and cardiovascular prognosis: From the partners coronary computed tomography angiography registry. *Cardiovasc. Diabetol.* **2017**, *16*, 14.
16. Hamer, M.; Johnson, W.; Bell, J.A. Improving risk estimates for metabolically healthy obesity and mortality using a refined healthy reference group. *European Journal of Endocrinology* **2017**, *177*, 169-174.
17. Fujihara, K.; Matsubayashi, Y.; Yamamoto, M.; Osawa, T.; Ishizawa, M.; Kaneko, M.; Matsunaga, S.; Kato, K.; Seida, H.; Yamanaka, N.*, et al.* Impact of body mass index and metabolic phenotypes on coronary artery disease according to glucose tolerance status. *Diabetes Metab.* **2017**, *43*, 543-546.
18. Yang, H.K.; Han, K.; Kwon, H.S.; Park, Y.M.; Cho, J.H.; Yoon, K.H.; Kang, M.I.; Cha, B.Y.; Lee, S.H. Obesity, metabolic health, and mortality in adults: A nationwide population-based study in korea. *Sci. Rep.* **2016**, *6*, 30329.
19. Park, Y.M.; Steck, S.E.; Fung, T.T.; Zhang, J.; Hazlett, L.J.; Han, K.; Merchant, A.T. Mediterranean diet and mortality risk in metabolically healthy obese and metabolically unhealthy obese phenotypes. *Int. J. Obes.* **2016**, *40*, 1541-1549.
20. Mark Park, Y.M.; Fung, T.T.; Steck, S.E.; Zhang, J.; Hazlett, L.J.; Han, K.; Lee, S.H.; Merchant, A.T. Diet quality and mortality risk in metabolically obese normal-weight adults. *Mayo Clin. Proc.* **2016**, *91*, 1372-1383.
21. Kim, N.H.; Seo, J.A.; Cho, H.; Seo, J.H.; Yu, J.H.; Yoo, H.J.; Kim, S.G.; Choi, K.M.; Baik, S.H.; Choi, D.S.*, et al.* Risk of the development of diabetes and cardiovascular disease in metabolically healthy obese people. *Medicine (United States)* **2016**, *95*.
22. Kaur, A.; Johnston, D.G.; Godsland, I.F. Does metabolic health in overweight and obesity persist? - individual variation and cardiovascular mortality over two decades. *European Journal of Endocrinology* **2016**, *175*, 133-143.
23. Sung, K.C.; Ryu, S.; Cheong, E.S.; Kim, B.S.; Kim, B.J.; Kim, Y.B.; Chung, P.W.; Wild, S.H.; Byrne, C.D. All-cause and cardiovascular mortality among koreans: Effects of obesity and metabolic health. *Am. J. Prev. Med.* **2015**, *49*, 62-71.
24. Panahi, M.H.; Yavari, P.; Khalili, D.; Mehrabi, Y.; Hadaegh, F.; Azizi, F. Obesity paradox: Incidence of coronary heart disease in patients with chronic kidney disease and metabolic syndrome. *Iranian Journal of Epidemiology* **2015**, *11*, 72-81.

25. Luo, D.; Liu, F.; Li, X.; Yin, D.; Lin, Z.; Liu, H.; Hou, X.; Wang, C.; Jia, W. Comparison of the effect of 'metabolically healthy but obese' and 'metabolically abnormal but not obese' phenotypes on development of diabetes and cardiovascular disease in chinese. *Endocrine* **2015**, *49*, 130-138.
26. Keihani, S.; Hosseinpanah, F.; Barzin, M.; Serahati, S.; Doustmohamadian, S.; Azizi, F. Abdominal obesity phenotypes and risk of cardiovascular disease in a decade of follow-up: The tehran lipid and glucose study. *Atherosclerosis* **2015**, *238*, 256-263.
27. Hamer, M.; Stamatakis, E. Metabolically healthy obesity and risk of all-cause and cardiovascular disease mortality. *J. Clin. Endocrinol. Metab.* **2012**, *97*, 2482-2488.
28. Berstein, L.M. Dark and light side of obesity: Mortality of metabolically healthy obese people. *Expert Review of Endocrinology and Metabolism* **2012**, *7*, 629-632.
29. Berstein, L.M. Dark and light side of obesity: Mortality of metabolically healthy obese people. *Expert Rev. Endocrinol. Metab.* **2012**, *7*, 629-632.
30. Calori, G.; Lattuada, G.; Piemonti, L.; Garancini, M.P.; Ragogna, F.; Villa, M.; Mannino, S.; Crosignani, P.; Bosi, E.; Luzi, L.*, et al.* Prevalence, metabolic features, and prognosis of metabolically healthy obese italian individuals: The cremona study. *Diabetes Care* **2011**, *34*, 210-215.

Table S1. Newcastle−Ottawa scale for assessment of quality of included cohort studies.

Study	Selection				Comparability		Outcome			**Total quality score**
Quality assessment criteria	Representativeness of exposed cohort	Selection of non−exposed cohort	Ascertainment of exposure	Demonstration that outcome of interest was not present at start of study	Adjust for the most important risk factors	Adjust for other risk factors	Assessment of outcome	Follow−up length	Loss to follow−up rate	
Acceptable (★)	Representative of general adult population in community (age/sex/being at risk of disease)	Drawn from the same community as exposed cohort	Secure records, Structured interview	Yes, or excluded when analysis	Yes, at least for age and sex	Yes, and smoking must be included	Independent blind assessment, record linkage	Follow−up >5 years	Follow−up completed, or small subjects lost (<20%), or lost subjects unlikely to introduce bias	

Kip, et al 2004[1]	★	★	★	★	★	–	–	–	–	**5**
Katzmarzyk, et al 2005[2]	★	★	★	★	★	★	★	★	★	**9**
St-Pierre, et al 2005[3]	★	★	★	★	★	★	★	★	★	**9**
Katzmarzyk, et al 2006[4]	★	★	★	★	★	–	★	★	★	**8**
Meigs, et al 2006[5]	★	★	★	★	★	★	★	★	★	**9**
Song, et al 2007[6]	★	★	★	★	★	★	★	★	★	**9**
Kuk, et al 2009[7]	★	★	–	–	★	★	★	★	–	**6**
Ärnlöv, et al 2010[8]	★	★	★	★	★	★	★	★	★	**9**
Flint, et al 2010[9]	★	★	–	★	★	★	★	★	–	**7**
Hosseinpanah, et al 2011[10]	★	★	★	★	★	★	★	★	★	**9**
Voulgari, et al 2011[11]	★	★	★	★	★	★	★	★	★	**9**
Bo, et al 2012[12]	★	★	★	–	★	★	★	★	★	**8**
Durward, et al 2012[13]	★	★	★	–	★	★	★	★	★	**8**
Ogorodnikova, et al 2012[14]	★	★	★	★	★	★	★	★	–	**8**
Appleton, et al 2013[15]	★	★	★	★	★	★	–	★	–	**7**
Choi, et al 2013[16]	★	★	★	★	★	★	★	★	–	**8**
Hanks, et al 2013[17]	★	★	★	★	★	★	★	★	–	**8**
Hinnouho, et al 2013[18]	★	★	★	–	★	★	★	★	–	**7**
Ortega, et al 2013[19]	★	★	★	★	★	★	★	★	–	**8**
Aung, et al 2014[20]	★	★	★	★	★	★	★	★	★	**9**
Morkedal, et al 2014[21]	★	★	★	★	★	★	★	★	–	**8**
Thomsen, et al 2014[22]	★	★	★	★	★	★	★	–	–	**7**
van der, 2014[23]	★	★	★	–	★	★	★	★	★	**8**
Hinnouho, et al 2015[24]	★	★	★	★	★	★	★	★	★	**9**
Mirbolouk, et al 2015[25]	★	★	★	–	★	★	★	★	★	**8**
Twig, et al 2015[26]	★	★	★	★	★	★	★	★	–	**8**

Cheng, et al 2016[27]	★	★	★	−	★	★	★	★	−	**7**
Dhana, et al 2016[28]	★	★	★	★	★	★	★	★	−	**8**
Guo, et al 2016[29]	★	★	★	★	★	★	★	★	−	**8**
Caleyachetty, et al 2017[30]	★	★	★	★	★	★	★	★	−	**8**
Doustmohamadian et al 2017[31]	★	★	★	−	★	★	−	★	★	**7**
Hansen, et al 2017[32]	★	★	★	★	★	★	★	★	−	**8**
Loprinzi, et al 2017[33]	★	★	★	−	★	★	★	★	−	**7**
Mirzaei, et al 2017[34]	★	★	★	★	★	★	★	★	★	**9**
Al−khalidi, et al 2018[35]	★	★	★	★	★	★	★	★	★	**9**
Eckel, et al 2018[36]	★	★	−	★	★	★	★	★	★	**8**
Kuk, et al 2018[37]	★	★	★	★	★	★	★	★	★	**9**
Lassale, et al 2018[38]	★	★	★	★	★	★	★	★	★	**9**
Lee, et al 2018[39]	★	★	★	−	★	−	★	−	★	**6**
Li, et al 2018[40]	★	★	★	★	★	★	−	−	−	**6**
Xu, et al 2018[41]	★	★	★	★	★	★	★	★	★	**9**
Zhang, et al 2018[42]	★	★	★	★	★	★	★	★	−	**8**

Each star represents if individual criterion within the subsection was fulfilled. In terms of representativeness, articles were awarded one star if the subpopulation with MHO represented the average adult in community. In terms of selection, one star was given if the non-exposed cohort; i.e., MHNW individuals; was drawn from the same community as MHO. One star was given if MHO was identified from secured records or structured interviews, and one star was given if the outcome of interest was not presented at the start of study or was excluded from analysis. In terms of comparability, one star was given if the article adjusted for the most important risk factor, age and sex. Smoking is a well-known confounding factor of BMI, CVD and all-cause mortality; thus, one star was given if the article adjusted for smoking habits. In terms of outcomes, one star was given if the CVD and mortality outcomes were obtained from independent blind assessment or record linkage. One star was given if follow-up was continued for more than five years[43], which was considered sufficient time for the development of CVD. It was difficult to assess the dropout rate of secondary cohort studies, and so we gave articles one star if all participants completed follow-up, only a small number was lost (<20%) or if the loss of participants was unlikely to introduce bias. If the original cohort was specified and the follow-up process and final results were clearly presented, we concluded that the quality was good.

Table S2. Characteristics of included cohort studies.

Study	Participants	Definition of obesity, BMI categories (kg/m²)	Definition of metabolic healthy / unhealthy	Adjusted variables	Diagnostic criteria and main results presented by MHO/MHOW compared with MHNW with HR (95%CI)	NOS
Kip, et al 2004[1]	US, WISE study, N=780, 100% women 9.6% MHO, 21-86 y/o, 3.5 yrs f/u	Measured; NW: <25; OW:25-29.9; OB: ≥30	ATP-III criteria	Age, race, prior MI, COPD, history of HF, number of lesions with ≥50% stenosis, PA	MACE (death, nonfatal MI, stroke, HF) by annual telephone or mail contact<br>All-cause mortality OW: 0.83(0.15; 4.63); OB: 0.66 (0.07; 6.01)<br>MACE OW: 0.76 (0.23, 2.56), OB: 0.74 (0.19; 2.84)	5
Katzmarzyk, et al 2005[2]	US, ACLS, N=19,173, 0 % women, 5.3% MHO 43.1 y/o, 10.2 yrs f/u	Measured; NW: 18.5-<25; OW:25-29.9; OB: ≥30	ATP-III criteria	Age, year of examination, smoking, alcohol, possible existence of CVD, parental history of premature CVD	Deaths identified by Social Security Death Index, death certificates, National Death Index; CVD mortality cause ICD-9 390–449.9<br>CVD mortality: OW: 1.27 (0.83–1.94), OB: 2.70 (1.40; 5.19)<br>All-cause mortality: OW: 0.94 (0.75; 1.17); OB: 1.31 (0.86; 2.01)	9
St-Pierre, et al 2005[3]	Canada, QCS, N=1,824, 0 % women, 24.5% MHO 34-64 y/o, 13 yrs f/u	Measured; NW: <25; OB: ≥30	TG, HDL-C, LDL, Apo B, IR, BP, hs-CRP	Age, smoking, medication use	First IHD included typical angina, coronary insufficiency (by standard FHS ECG criteria), nonfatal MI, coronary death<br>Fatal + non-fatal CHD incidence 1.53 (0.79; 3.00)	9
Katzmarzyk	US, ACLS and	Measured;	ATP-III criteria, IDF	Age, year of	Deaths identified by death certificates,	8

, et al 2006[4]	NHANES, N=20,789, 0 % women, 18.4% MHO 43.3±9.7 y/o, 11.4 yrs f/u	NW: <90cm; OB: >102cm		examination	National Death Index; CVD mortality cause ICD-9 390–449.9<br>CVD mortality OR =1.40<br>All-cause mortality OR =1.26	
Meigs, et al 2006[5]	US, FOS, N=2,902, 55% women, 8.1% MHO, 53 y/o, 11.4 yrs f/u	Measured; NW: <25; OW:25-29.9; OB: ≥30	ATP-III criteria	Age, sex, smoking, LDL-C	By standard FHS ECG criteria<br>Fatal + non-fatal CVD incidence<br>OW: 1.30(0.89; 1.90), OB 1.48 (0.87; 2.55)	9
			MUH if HOMA-IR (mole×μU/L^2) value belongs to the top quartile		Fatal + non-fatal CVD incidence<br>OW: 1.25(0.86; 1.81), OB: 1.42 (0.87; 2.33)	
Song, et al 2007[6]	US, WHS, N=25,626, 100 % women, 11.4% MHO 39-89 y/o, 10.2 yrs f/u	Measured; NW: <25; OW:25-29.9; OB: ≥30	Modified ATP-III criteria (without WC)<br><br>MH if ≤2 criteria (incident diabetes during follow-up, substituted for FBG)	Age, randomized treatment assignment (aspirin and vitamin E), smoking, exercise, alcohol, total calorie intake, postmenopausal hormone use, multivitamin use, parental history of MI before 60 years	Diagnoses confirmed by a committee of cardiologists and one Neurologist<br>Fatal + non-fatal CVD incidence<br>OW: 0.91 (0.70; 1.18); OB: 1.05 (0.66; 1.66]	9

Kuk, et al 2009[7]	US, NHANES III, N=6,011<br>1.3% MHO<br>52 % women in MHO,<br>45 y/o,<br>8.7 yrs f/u	Self-reported, NW: 18.5-25; OB: ≥30	MUH if ≥1 criterion of:<br>1) TG ≥1.69 mmol/l or medications; 2) BP≥130/85 mmHg, or medications; 3) glucose ≥ 5.6 mmol/l or medications; 4) HDL-C<1.04 mmol/l (men), <1.29 mmol/l (women); 5) HOMA-IR ≥2.5 (mole×μU/L^2)	Age, sex, income, ethnicity, smoking, alcohol	Link to Public Access Mortality<br>All-cause mortality: OW: 0.45 (0.2; 1.2), OB: 2.8 (1.2; 6.7)	6
			MUH if HOMA-IR ≥2.5 (mole×μU/L^2) and ≥1 criteria of: 1) TG ≥ 1.69 mmol/l or medications; 2) BP≥130/85 mmHg, or medications; 3) glucose ≥ 5.6 mmol/l or medications, 4) HDL-C<1.04 mmol/l (men), <1.29 mmol/l (women)		All-cause mortality OW: 0.91(0.5; 1.8), OB: 2.58 (1.0; 6.7)	

Ärnlöv, et al 2010[8]	Sweden,<br>N=1,758,<br>0% women<br>1.7% MHO<br>50 y/o<br>30 yrs f/u	Measured;<br>NW: <25;<br>OW:25-29.9; OB: ≥30	Modified ATP-III criteria (BMI ≥29.4 substituted for WC)	Age, smoking, LDL-C	Based on ICD-8, ICD-9, ICD-10<br>Fatal and hospitalized CVD incidence<br>OW: 1.52 (1.28; 1.80), OB: 1.95 (1.14; 3.34)<br>CV mortality: OW: 1.44 (1.14; 1.83), OB: 1.20 (0.49; 2.93)<br>All-cause mortality: OW: 1.21 (1.03; 1.40), OB: 1.65(1.03; 2.66)	9
			MUH if HOMA-IR >3.43(mole×μU/L^2) (based on the top quartile)		Fatal and hospitalized CVD incidence:<br>OW: 1.44 (1.18; 1.77), OB: 1.91 (1.07-3.41)<br>CV mortality: OW:1.36 (1.02; 1.80), OB: 1.80 (0.79; 4.08)<br>All-cause mortality: OW: 1.22 (1.02; 1.46), OB: 2.04 (1.25; 3.32)	
Flint, et al 2010[9]	US, HPFS, N=42,351, 0% women, NA % MHO, 53.6 y/o, 16 yrs f/u	Self-reported;<br>NW:18.5-22.9, high NW: 23.0-24.9; OW I: 25-26.9;<br>OW II: 27.0-29.9; OB:	No hypercholesterolemia, hypertension or diabetes	For both sex: age, family history of MI, smoking, height, marital status, intake of alcohol, saturated fat, polyunsaturated fat, trans fat, folate, vitamin E, and total energy	CHD defined as MI, using WHO criteria for MI; death ascertained by national death index and by questionnaire<br>CHD<br>High NW: 1.07 (0.90; 1.26), OW I: 1.44 (1.22; 1.70), OW II: 1.76 (1.48; 2.10), OB: 1.95 (1.57; 2.42)	7

	US, NHS study, N=76,703, 100% women, NA % MHO,53.9 y/o, 16 yrs f/u	≥30		The above and HRT use	CHD High NW: 1.08 (0.93; 1.26), OW I: 1.41 (1.21: 1.65), OW II: 1.54 (1.31; 1.81), OB, 2.14 (1.83; 2.50)	
Hosseinpanah, et al 2011[10]	Iran, TLGS, N=6,215 56.9 % women 6.6 % MHO 44.6± 0.5y/o in MHO, 8.1 yrs f/u	Measured; NW: 18.5-24.9; OW: 25.0-29.9; OB: ≥30	IDF (cut-point WC ≥89 cm for men, ≥91 cm for women)	Age, sex, exercise, smoking, family history of premature CHD, high TC	CVD defined as any CHD events (included MI, angiographically proved CHD, CHD death), stroke (a new neurological deficit lasted ≥24 h) or CVD death<br>Fatal + non-fatal CVD incidence: OW: 1.10 (0.76: 1.61), OB: 1.07 (0.59; 1.96)	9
Voulgari, et al 2011[11]	Greece, N= 550, 31 % MHO 53.2 % women in MHO, 60 y/o, 6 yrs f/u	Measured; NW: <25; OW: 25.0-29.9; OB: ≥30	ATP-III criteria	Age, sex, IGT, dyslipidemia, HTN, smoking, physical inactivity, LVH, function on echocardiography	Identified by physician on the basis of symptoms, signs, prescription of medication and objective evidence of structural or functional heart disease by echography<br>Fatal + non-fatal HF incidence: OW: 1.12(0.35; 1.33); OB: 0.41 (0.1; 1.31)	9
Bo, et al 2012[12]	Italy, N=1,658, 22 % MHO, 66.7% women in MHO, 45-64 y/o, 9 yrs f/u	Measured; NW: <25; OW: 25.0-29.9; OB: ≥30	MUH if HOMA-IR > 2.5 (mole×μU/L^2) (approximately upper quartile in the cohort)	Age, sex, smoking	CVD: Documented by family physician (angina, MI, CHD, TIA, stroke, gangrene, amputation, vascular surgery, HF); CV death by ICD 410–414, 430–438, 440, 390–459 and 798.1<br>CVD incidence: OW: 1.18 (0.56; 2.50),	8

					OB: 2.76 (1.05; 7.28)<br>CVD mortality: OW: 1.52 (0.52; 2.52),<br>OB: 2.95 (1.03; 3.98)<br>All-cause mortality OW: 0.98 (0.41; 1.55),<br>OB: 1.57 (0.93; 2.21)	
Durward, et al 2012[13]	US, NHANES-III,<br>N=4373,<br>49.4 % women,<br>3.4% MHO<br>37.1 ± 10.9 y/o,<br>14.7 yrs f/u	Measured; NW: 18.5-24.9; OW: 25.0-29.9; OB: ≥30	HOMA-IR ≥2.5 (mole×μU/L^2)	Sex, age, income, education, race/ethnicity, smoking, alcohol, marital status, leisure time PA, menopausal status	Linked to mortality files<br>All-cause mortality: OB: 1.42 (0.6; 3.2)	8
			ATP-III criteria		All-cause mortality: OB: 1.54 (0.7; 3.3)	
			MUH:>2 of: 1) HOMA-IR (mole×μU/L^2) <1.95 or medications; 2) TG≥1.7 mmol/L; 3) HDL-C <1.04 mmol/L (men), <1.30 mmol/L (women); 4) LDL-C ≥2.6 mmol/L; 5) TC ≥5.2 mmol/L or medications		All-cause mortality: OB: 1.48 (0.5; 4.2)	
Ogorodnikova, et al 2012[14]	US, ARIC/CHS study,<br>N=17,544,<br>67.8% women in MHO,<br>55.5 y/o,<br>11.8 yrs f/u	Measured; NW: 18.5-24.9; OW: 25.0-29.9; OB: ≥30	ATP-III criteria without WC criterion) MHO = 6.7%	Age, sex, race, smoking, alcohol	Review of death certificates and hospital discharge records<br><br>Fatal + non-fatal CVD (CHD and stroke) incidence: OB: 1.24 (0.99; 1.57)	8
			Expanded ATP-III criteria, MHO = 4.5%			
			MUH if HOMA-IR (mole×μU/L^2) value			

			belongs to the top quartile, MHO = 5.0%			
Appleton, et al 2013[15]	Australia, NWAHS, N=3,743, 12.1% MHO, 14.4 % women in MHO, ≥18 y/o, 8.2 yrs f/u	Measured; NW: 18.5-24.9; OW:25-29.9; OB: ≥30	IDF (without WC) criteria	Age, sex, smoking, household income, education, PA, LDL-C	Self-reported data<br><br>Non-fatal CVD incidence: OW:1.17(0.68:2.02); OB:1.16 (0.58; 2.29)	7
Choi, et al 2013[16]	Korea, SWS study, N=2,317, 9.7% MHO, 77.9 % women, 69.8 ± 5.6 y/o, 10.3(median) yrs f/u	Measured; NW: <23; OW:23-25; OB: ≥25	Modified ATP-III criteria (without WC criterion; using 2 hPG≥7. 8 mmol/l or treatment for DM instead of FBG≥5. 6 mmol/l)	Age, sex, smoking, alcohol, presence of DM, HTN, CVD	Death certificate data from Korean National Statistical Office<br>CV death by ICD-10 I00-I79<br><br>Compared to MHOW, MHO CVD 1.78(0.94; 3.4), all-cause mortality 1.18(0.84; 1.68)	8
Hanks, et al 2013[17]	US, REGARDS study, N=4374 with CKD, 53 % women, 9.7% MHO 69±10 y/o, 4.5 yrs f/u	Measured; NW: 18.5-24.9; OW:25-29.9; OB: ≥30	JIS criteria	Age, sex, region, PA, smoking, history of CHD/stroke, education, family income, urinary albumin to creatinine ratio, eGFR	Linked to Social Security Death Index, death certificates, National Death Index<br><br>All-cause mortality: OW:0.74(0.57-0.96); OB: 0.78 (0.55; 1.10)	8

Hinnouho, et al 2013[18]	UK, Whitehall II cohort study, N=5,269, 28.3% women, 4.5% MHO, 41-63 y/o, 17.7 yrs f/u	Measured; NW: 18.5-24.9; OW:25-29.9; OB: ≥30	ATP-III criteria	Age, sex, occupational grade, PA, smoking, alcohol, fruit, vegetable consumption, marital status, ethnicity	Defined by ICD-9 or ICD-10 codes<br>CVD mortality: OW:1.03(0.55-1.91); OB:2.49 (1.05; 5.91)<br>All-cause mortality: OW:0.94(0.69-1.27); OB:1.81 (1.16; 2.84)	7
			MUH if HOMA-IR (mole×μU/L^2) >1.7 (men) or >1.52 (women) (based on quartiles)		CVD mortality: OW:1.16(0.74-1.80); OB:1.04 (0.41; 2.66)<br>All-cause mortality: OW:0.96(0.77-1.24); OB:1.08 (0.67; 1.74)	
Ortega, et al 2013[19]	US, ACLS, N= 43,265, 24.3 % women, 4.0% MHO 44.2 ± 9.9 y/o, 14.3(6.0-19.8) yrs f/u	Measured; NW: <25; OW:25-29.9; OB: ≥30	JIS criteria	Age, sex, examination year, smoking, alcohol consumption, parental history of CVD	CV mortality by ICD-9,10, non-fatal CVD by diagnosis of MI, stroke, or a coronary revascularization procedure ascertained from mail-back health surveys<br>Non-fatal CVD events: OB: 1.72 (1.33; 2.12)<br>CVD mortality: OB: 2.44 (1.90; 2.97)<br>All-cause mortality; OB: 1.54 (1.24; 1.84)	8
Aung, et al 2014[20]	US, San Antonio Heart Study, N=3,700, 10.3% MHO 41.8 % women in MHO, 25-64 y/o, 7.4(6.3-10.3) yrs	Measured; NW: <25; OW:25-29.9; OB: ≥30	MUO if >1 of the criteria: 1) TG ≥1.7 mmol/L; 2) BP ≥130/85 mmHg or medication; 3) FBG ≥5.6 mmol/L or medication; 4) HDL-C <1.04 (men); 1.29	Age, sex, ethnic origin, smoking	Self-reported MI, stroke, or coronary revascularization procedure or any CV death by ICD-9 390-459<br><br>Fatal + non-fatal CVD incidence: OW:2(1.0-4.1); OB:4.40 (2.0; 9.5)	9

	f/u		mmol/L (women) or medication; 5) HOMA-IR (mole×μU/L^2)> 90th percentile			
Morkedal, et al 2014[21]	Norway, HUNT study, N=61,299, 5.7% MHO, 68.9% women in MHO, ≥20 y/o, 12 yrs f/u	Measured; NW: <25; OW:25-29.9; OB: ≥30	Modified IDF criteria (WC >94 cm for men, >80 for women or BMI > 30 and two of the other criteria)	Age, sex, smoking, time since last meal, education, marital status, PA, alcohol consumption	Diagnosed by cardiologists according to ESC/ACC consensus guideline<br>MI incidence: OW:1.3 (1.1-1.5); OB:1.10 (0.9; 1.4)<br>HF incidence: OW:1.0 (0.8-1.3); OB: 1.7 (1.3; 2.3)	8
Thomsen, et al 2014[22]	Denmark, CGPS, N=71,529, 6.2% MHO, 68 % women in MHO, 20-100 y/o, 3.6 yrs f/u	Measured; NW: 18.5-24.9; OW:25-29.9; OB: ≥30	Modified JIS criteria (without WC; FBG substituted by non-FBG ≥200 mg/dL)<br><br>MH if <3 criteria	Age, sex, smoking, LDL-C, lipid-lowering medication, aspirin use, physical inactivity	IHD based on ICD-8: 410-414, ICD-10: I20-I25<br>Fatal + non-fatal IHD incidence: OW: 1.08(0.95-1.24); OB:1.45 (1.20; 1.77)<br>Fatal + non-fatal MI incidence: OW: 1.26 (1.00-1.61); OB:1.88 (1.34; 2.63)	7
van der, et al 2014[23]	Netherlands, EPIC-MORGEN study, N=20299, 4.4% of MHO, 73.9 % women in MHO, 20-59 y/o, 13.4 yrs f/u	Measured; WC: Normal waist (<94 cm for men, <80 cm for women); Abdominal	MH if no metabolic risk factors: 1) BP ≥130/85 mmHg or medication; 2) TC ≧ 6.5mmol/L, or HDL-C <1.03 mmol/L (men),	Age, sex, smoking, education, PA, total energy, protein, carbohydrate intake	Vital status was obtained through linkage with the municipal administration registries<br><br>By WC: All-cause mortality OW:1.09(0.73-1.60); OB:1.66 (1.11; 2.49)<br>By BMI: All-cause mortality 1.63 (0.90;	8

		OW (94-102cm for men; 80-88 cm for women); Abdominal OB (≧ 102cm for men, 88 cm for women) BMI : OB ≥30	<1.29 mmol/L (women) or medication; 3) NFBG ≧ 7.8mmol/L or FBG≥5.6 mmol/L or self-reported DM		2.95)	
Hinnouho, et al 2015[24]	UK, Whitehall II study, N=7,122, 3.9% MHO 30.3 % women, 39-63 y/o, 17.5 yrs f/u	Measured; NW: 18.5-24.9; OW:25-29.9; OB: ≥30	ATP-III criteria (without WC), MH if ≤1 criterion	Sex, socioeconomic, marital status, ethnicity, PA, smoking, alcohol, fruits /vegetables consumption, CVD medication and procedures	CVD events included fatal CHD, by ICD-9: 410–414 or ICD-10: I20–25<br><br>Fatal + non-fatal CVD incidence: OW:1.24(1.01-1.51); OB:1.95 (1.37; 2.77)	9
Mirbolouk, et al 2015[25]	Tehran, TLGS, N= 1199, 18.8% MHO 62.8 % women in MHO,	Measured; NW: 18.5-24.9; OW:25-29.9; OB:	JIS criteria	Age, sex, smoking, TC and lipid lowering drugs.	CVD defined as any CHD events (included MI, angiographic proven CHD), stroke (a new neurological deficit lasted >24 h) or CVD death<br>CVD events: OW:1.21(0.77-1.91); OB:	8

	≥65 y/o,<br>9.7 yrs f/u	≥30			1.48 (0.65; 3.39)<br>CVD mortality: OW:2.07(0.89-4.81); OB: 1.11 (0.13; 9.04)<br>All-cause mortality: OW:1.38(0.87-2.19); OB: 1.34 (0.51; 3.50)	
Twig, et al 2015[26]	Israel, MELANY cohort, N= 31,684, 0 % women, 1.9% MHO, 31.2 ± 5.7 y/o, 6.1 yrs f/u	Measured; NW: 18.5-24.9; OW:25-29.9; OB: ≥30	ATP-III criteria	Age, family history of CAD, LDL-C, WBC count, smoking status, PA	Based on diagnostic procedure treadmill exercise test, ST segment depression, symptoms of angina, coronary angiography<br><br>CHD incidence: OW:1.86(0.77-4.49); OB: 5.08 (1.69; 11.24)	8
Cheng, et al 2016[27]	US, GRAS cohort, N=4551, 58.4 % women 5.0% MHO<br>74 ± 4.7 y/o, 10.9(0.3–14.4) yrs f/u	Measured Desirable: 18.5-24.9; OW :25.0-29.9; OB class I: 30.0–34.9; OB class II/II: ≥35.0	MH if ≤1 of these criteria: 1) TG ≥1.69 mmol/L; 2) HDL <1.29 mmol/L (women) or <1.03 mmol/L(men); 3) BP≥130/85 mmHg or HTN diagnosis; 4) FBG≥5.56 mmol/L or DM diagnosis	Age, sex, smoking, alcohol, hypercholesterolemia drug, LDL-C, disease burden	Deaths identified by medical records and Social Security Death Index data<br><br>All-cause mortality<br>OW: 0.90(0.73-1.13)<br>OB class I: 0.58 (0.42; 0.80)<br>OB class II/III: 0.78 (0.48; 1.27)	7
Dhana, et al 2016[28]	Netherlands, Rotterdam Study, N=5314, 0% MHO	Measured NW:18.5-25; OW 25–29.9; OB:	JIS criteria	Age, sex, smoking, TC level, lipid-lowering medication use, GFR, alcohol,	Fatal CHD events coded by the definitions applied within the CHS and ARIC study	8

	60.3 % women, 68 y/o, 10.3 (8.1-11.7) yrs f/u	≥30		education, PA	CVD: OW:1.08(0.89-1.32); OB:1.07(0.75; 1.53)	
Guo, et al 2016[29]	US, ARIC/ CARDIA study: N= 14,685, 54.4 % women, 1.8% MHO, 54.3 y/o, 18.7 yrs f/u	Measured; NW: 18.5-24.9; OB: ≥30	Modified ATP-III criteria	Age (log transformed), sex, race, income, education, smoking, drinking	Review of death certificates and hospital discharge records<br><br>CHD: OB:1.09 (0.47; 1.70)<br>Mortality 1.03 (0.69; 1.37)	8
Caleyachetty, et al 2017[30]	UK, N=3495777, 14.8% MHO 41.6 % women in MHO, 42.6 ±13.8 y/o in MHO, 5.4 yrs f/u	Record from database. NW: 18-<25; OW: 25- <30; OB: ≥30	MH if none of the following criteria: DM, HTN or prescription of lipid-lowering agents	Age, sex, smoking, social deprivation	According to the database<br>CVD includes: CHD, angina, IHD, MI, stroke, TIA, HF, PAOD<br>CHD: OW:1.30(1.27;1.34); OB: 1.49(1.45; 1.54)<br>Stroke: OB:1.07(1.04; 1.11)<br>HF: OW:1.11(1.06;1.16); OB: 1.96(1.86; 2.06)<br>PAOD: OW:0.92(0.88-0.96); OB: 0.91 (0.86; 0.96)	8
Doustmohamadian, et al 2017[31]	Iran, TLGS study, N=8804, 54.8% women, 12.8% MHO, 47.7±12.6 y/o, 12(8.7-12.5) yrs f/u	Measured WC≥89 cm for men, WC≥91 cm for women	JIS criteria<br>MH if ≦2 of the criteria	Age, sex, smoking, education, PA	Not mentioned<br>All-cause mortality 1.35(0.89; 2.03)	7

Hansen, et al 2017[32]	Denmark, Inter99 study, N=6238, 51.1 % women, 2.8% MHO, 46.3 y/o in men, 10.6 yrs f/u	Measured NW:18.5-24.9; OW:25-29.9; OB≧30	MH if none of the following criteria: 1)BP≥ 140/90mmHg; 2) TG ≥1.7 mmol/L; 3) FBG ≥6.1 mmol/L; 4) HDL <1.0 mmol/L (men), <1.2 mmol/L (women)	Age, intervention, smoking, PA, diet, cohabitation, ethnicity	IHD by ICD-10 codes and relevant procedures (CABG, coronary thromboendarterectomy, revascularization, embolectomy)<br><br>IHD: OW:1.5(0.8;3); OB: 1.8 (0.7; 4.8) for women, OW:1.1(0.5;2.4); OB: 3.1 (1.1; 8.2) for men	8
Loprinzi, et al 2017[33]	USA, NHANES, N=7579, 51% women, 3.9% MHO, 45.9 y/o, 103 months f/u	Measured; NW: 18.5-24.9; OW: 25.0–29.9; OB: ≥30	ATP-III criteria	Age, sex, ethnicity, smoking, PA	Deaths were identified by Social Security Death Index, death certificates, National Death Index<br>Non-CV mortality (CHD, HF or heart attack were excluded):<br>OW:2.30(1.37;3.85); OB: 2.59 (1.02; 6.56)<br>All-cause mortality: OW:1.88(1.08;3.28); OB: 2.41 (1.07; 5.46)	7
Mirzaei, et al 2017[34]	Iran, TLGS, N=7842<br>55.2% women, 2.0% MHO<br>41.8±9.3 y/o in MHO,<br>11.9 yrs f/u	Measured; NW: 18.5-24.9; OW: 25.0-29.9; OB: ≥30	JIS criteria	Age, sex, smoking, education, PA, family history of premature CHD, TC	CVD events: defined as any CHD, stroke (a new neurological deficit that lasted ≥24 h), or CVD death (fatal CHD or fatal stroke)<br>Incidence of CVD: OW:1.22(0.73;2.04); 1.74 (0.68; 4.44)	9
			Insulin sensitive: HOMA-IR < 2.6 (mole×μU/L^2); Insulin		Incidence of CVD: OW:1.70(1.13;2.55); OB: 1.96(1.18; 3.24)	

			resistant: HOMA-IR≥2.6 (mole×μU/L^2)			
Al-khalidi, et al 2018[35]	USA, NHANES III, N=11,333, 3.2% MHO, 100% women, 37.9 y/o in MHO, 19.6 yrs f/u	Measured; NW: 18.5-24.9; OW: 25.0–29.9; OB: ≥30	JIS criteria	Age, sex, ethnicity, smoking, educational, leisure-time PA, eGFR, serum CRP, 25(OH)D	Cause of mortality by ICD-10; CM mortality defined by deaths classified as diseases of heart, cerebrovascular diseases, DM<br>CM mortality: OW:0.94(0.38;2.33); OB: 1.21(0.33; 4.46)<br>Non-CM mortality: OW:0.84(0.56; 1.27); OB: 0.84 (0.47; 1.50)<br>All-cause mortality: OW:0.85(0.59; 1.22); OB: 0.89(0.52; 1.51)	9
Eckel, et al 2018[36]	USA, NHS, N=90257<br>6.4% MHO<br>100% women, 45.9 ±7.0 y/o in MHO,<br>24 yrs f/u	Measured; NW: 18.5-24.9; OW: 25.0–29.9 ; OB: ≥30	MH if none of the following criteria: self-reports of physician-diagnosed HTN, DM, hypercholesterolemia	Age, race, education, alcohol, smoking, post-menopausal status, HRT use, physical examinations for screening purposes, aspirin use, family history of MI, DM, PA	MI by WHO criteria: OW:1.13(0.92;1.37); OB: 1.44 (1.11; 1.86)<br>Stroke (CT or MRI showed a neurological deficit with sudden or rapid onset ≥ 24h): OW:1.29(1.05;1.58); OB: 1.37 (1.04; 1.81)<br>CVD (fatal and non-fatal MI + stroke : OW:1.20(1.04;1.39); OB:1.39(1.15; 1.68)	8
Kuk, et al 2018[37]	US, ACLS, N=54089, 1.2% MHO, 56.3 % women in MHO, 39.6 y/o in	Measured; NW: 18.5-<25; OW: 25.0–29.9; OB: ≥30	ATP-III criteria	Age, sex, ethnicity, smoking, f/u time	Using mortality linkage data file<br><br>Mortality risk: OB: 1.15(1.01; 1.32)	9

	MHO, 12.8 yrs f/u					
Lassale, et al 2018[38]	Europe, EPIC-CVD study, N=520,000, 7.2% MHO, 64 % women in MHO, 52.8 yrs in MHO, 12.2 yrs f/u	Measured; NW: 18.5-<25; OW: 25.0-29.9; OB: ≥30	IDF criteria	Age, sex, center, education, smoking, diet, PA	CHD (MI, IHD, angina pectoris) used self-report, linkage to care registers, admissions, and mortality data<br><br>CHD: OW:1.26(1.14;1.4); OB: 1.28 (1.03; 1.58)	9
Lee, et al 2018[39]	Korea, KAMIR-NIH, N=6935, 0% women, 18.7% MHO, 57.2±11.7 y/o in MHO,<br>1 yr f/u	Measured; NW:18.5-23 OB: ≥25	ATP III criteria	age, SBP, LVEF <40%, use of new P2Y12 inhibitors, statins, bare metal stents	MACE (death from any cause, MI, repeat PCI at target or non-target vessel revascularization, CABG) retrieved from hospital electronic medical records and/or by telephone interview.<br>All-cause mortality 0.89 (0.45; 1.76)<br>Cardiovascular death 1.28 (0.54; 3.02)<br>MACE 1.02 (0.75; 1.37)	6
Li, et al 2018[40]	China, Beijing cohort study, N=9,393, 65.9% women, 6.7% MHO 56.5 ± 7.5 y/o, 3.2 yrs f/u	Measured; NW: 18.5-<24; OW: 24.0–27.9; OB: ≥28	ATP-III criteria	Sex, age, income, education, PA, smoking, drinking, ideal diet, family history of CVD, LDL-C	CVD (admission for MI, coronary revascularization, HF or stroke)<br>using epidemiological questionnaire<br><br>CVD events: OW: 1.09(0.7;1.7); OB: 1.91(1.13; 3.24)	6

Xu, et al 2018[41]	China, Kailuan study, N=91,866, 7.3% MHO 19.9% women in MHO, 18-98 y/o, 8 yrs f/u	Measured; NW: 18.5-<24; OW: 24.0–27.9; OB: ≥28	IDF criteria	Age, sex, education, income, smoking, drinking, PA, sodium intake, LDL-C, hs-CRP, eGFR	MI and death due to MI identified from medical records and death certificates<br><br>MI: OW: 1.08(0.89;1.31); OB: 1.76(1.37; 2.25)	9
Zhang, et al 2018[42]	China, N=3,485, 0% women, 7% MHO, >60 y/o, 5 yrs f/u	Measured; NW: <24; OW: 24.0–27.9; OB: ≥28	MH if none of the following criteria: 1) HTN; 2) DM; 3) dyslipidemia	Age, smoking, HTN, DM, dyslipidemia	Mortality defined by ICD-10<br><br>All-cause mortality: OW:0.86(0.55;1.35); OB: 1.56 (0.85; 2.86)<br>CV mortality: OW:0.96(0.51;1.81); OB: 1.4 (0.56; 3.51)	8

AbOW, Abdominal overweight; AbOB, Abdominal obesity; ACC, American College of Cardiology; ACLS, Aerobics Center Longitudinal Study; ADL, activities of daily living; Apo B, Apolipoprotein B; ARIC study, Atherosclerosis Risk in Communities study; BMI, body mass index; BP, blood pressure; CABG, coronary artery bypass graft; CARDIA study, Coronary Artery Risk Development in Young Adults Study; CES-D, The Center for Epidemiologic Studies Depression Scale; CGPS, Copenhagen General Population Study; CHS, Cardiovascular Health Study; CHD, coronary heart disease; CHRLS, China Health and Retirement Longitudinal Study; CI, confidence interval; CKD, chronic kidney disease; CM, cardiometabolic; COPD, chronic obstructive pulmonary disease; CRP, C-reactive protein; CVD, cardiovascular disease; CT, computed tomography; CTA, cardiac computed tomography angiography; DM, Diabetes mellitus; ECG, electrocardiogram; eGFR, estimated glomerular filtration rate; ELSA, English Longitudinal Study of Ageing; EPIC-CVD study, European Prospective Investigation into Cancer and Nutrition study; ESC, European Society of Cardiology; FBG, fasting blood glucose; FHS, Framingham Heart Study; FOS, Framingham Offspring Study; f/u, follow up; GRAS, Geisinger Rural Aging Study; h, hour; HDDRISC, The Heart Disease and Diabetes Risk Indicators in a Screened Cohort; HDL-C, high-density lipoprotein cholesterol; HF, heart failure; HOMA-IR, homeostasis model assessment-insulin resistance; HPFS, Health Professionals Follow-up Study; HR, hazard ratio; HRT, hormone replacement therapy; HSCIC, NHS Information Centre for Health and Social Care; hs-CRP, high sensitivity C-reactive protein; HSE, Health Survey for England; HTN, hypertension; IADL, Independent activities of daily living; ICD,

International Classification of Disease; IDF, International Diabetes Federation; IR, insulin resistance; IGT, impaired glucose tolerance; IHD, ischemic heart disease; JIS, Joint Interim Statement; JPHC study, Japan Public Health Center-based Prospective study; KIHDRF study, Kuopio Ischemic Heart Disease Risk Factor Study; KoGES, Korean Genome and Epidemiology Study; L, liter; LDL-C, low-density lipoprotein cholesterol; LVEF, left ventricular ejection fraction; LVH, left ventricular hypertrophy; MACE, Major adverse cardiovascular event; MELANY cohort, Metabolic, Lifestyle and Nutrition Assessment in Young Adult Cohort; MESA study, Multi-Ethnic Study of Atherosclerosis study; MetS, metabolic syndrome; MH, metabolically healthy; MHOO, metabolically healthy overweight/obese; MI, myocardial infarction; MRI, magnetic resonance imaging; MUH, metabolically unhealthy; ATP III, National Cholesterol Education Program- Adult Treatment program III; MHNW, metabolically healthy normal weight; MHO, metabolically healthy obesity; MHOW, metabolically healthy overweight; MI, myocardial infarction; MUNW, metabolically unhealthy normal weight; MUO, metabolically unhealthy obesity; NA, not available; NHANES III, Third National Health and Nutrition Examination Survey; NHLBI, National Heart, Lung, and Blood Institute; NHS, Nurses' Health Study; NOS, Newcastle-Ottawa scale; NW, normal weight; NWAHS, North West Adelaide Health Study; OB, obese; OR, odds ratio; OW, overweight; PA, physical activity; PAOD, peripheral artery occlusion disease; PCI, percutaneous coronary intervention; QCS, Quebec Cardiovascular Study; REGARDS study, Reasons for Geographic and Racial Differences in Stroke study; SHS, Scottish Health Survey; SWS study, South-West Seoul Study; TC, total cholesterol; TG, triglycerides; TIA, transient ischemic attack; TLGS, Tehran Lipid and Glucose Study; UK, United Kingdom; US, United States; VF, ventricular fibrillation; VMCUN cohort, Vascular-Metabolic CUN cohort; VT, ventricular tachycardia; WBC, white blood cell; WC, waist circumstance; WHO, world health organization; WHR, waist-to-hip ratio; WHS, Women's Health Study; WISE, Women's Ischemia Syndrome Evaluation; y/o, years old; yrs, years

Figure S1 A Forest plot of cardiovascular disease, comparing participants with metabolically healthy obesity and participants with metabolically healthy normal weight, with a subgroup analysis by definitions of metabolic health

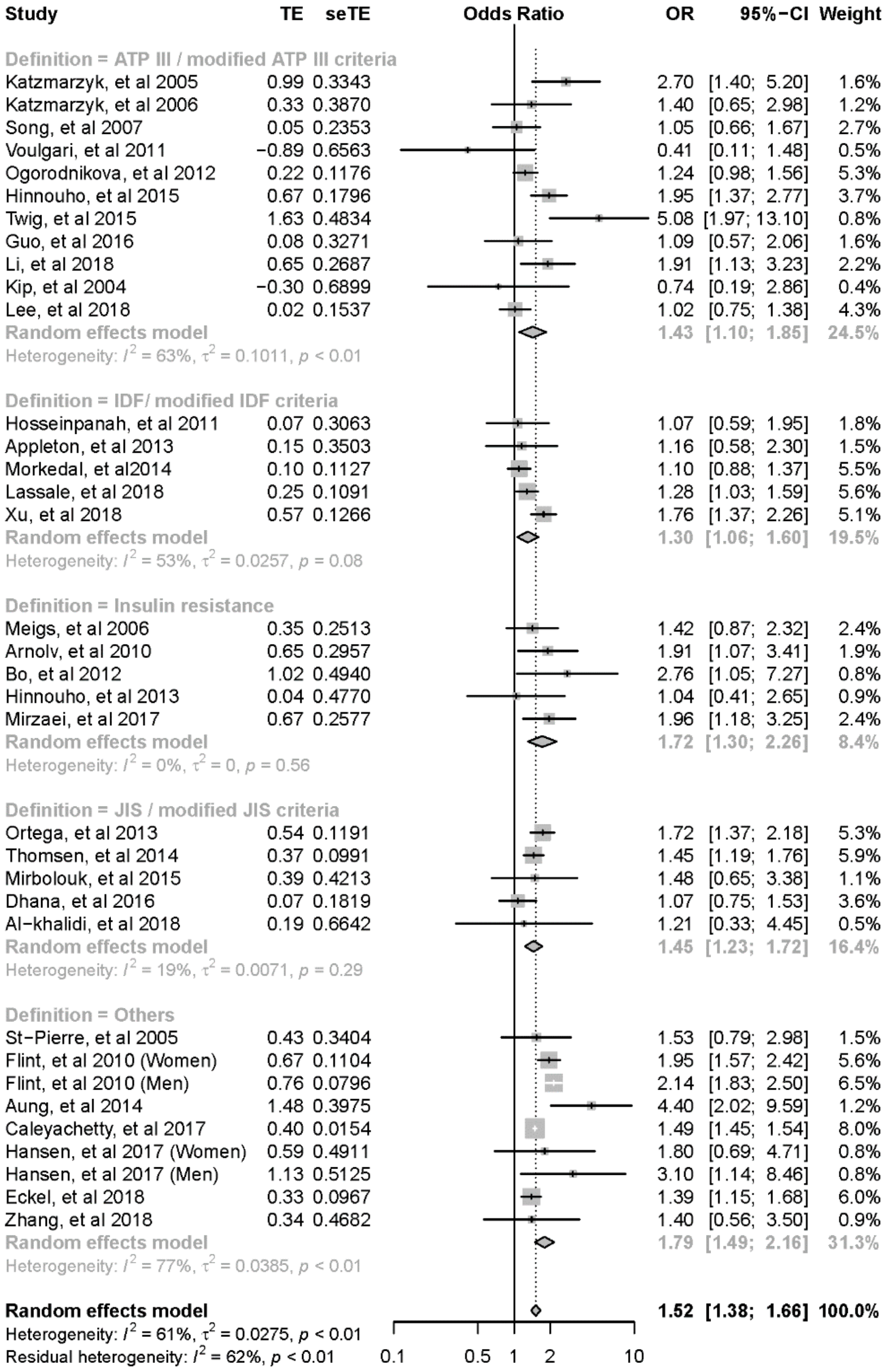

ATP III, Adult Treatment program III; CI, confidence interval; IDF, International Diabetes Federation; JIS, Joint Interim Statement; OR, odds ratio; SE, standard error; TE, treatment effect

Figure S2. A Forest plot of cardiovascular disease, comparing participants with metabolically healthy obesity and participants with metabolically healthy normal weight, with a subgroup analysis by definitions of obesity.

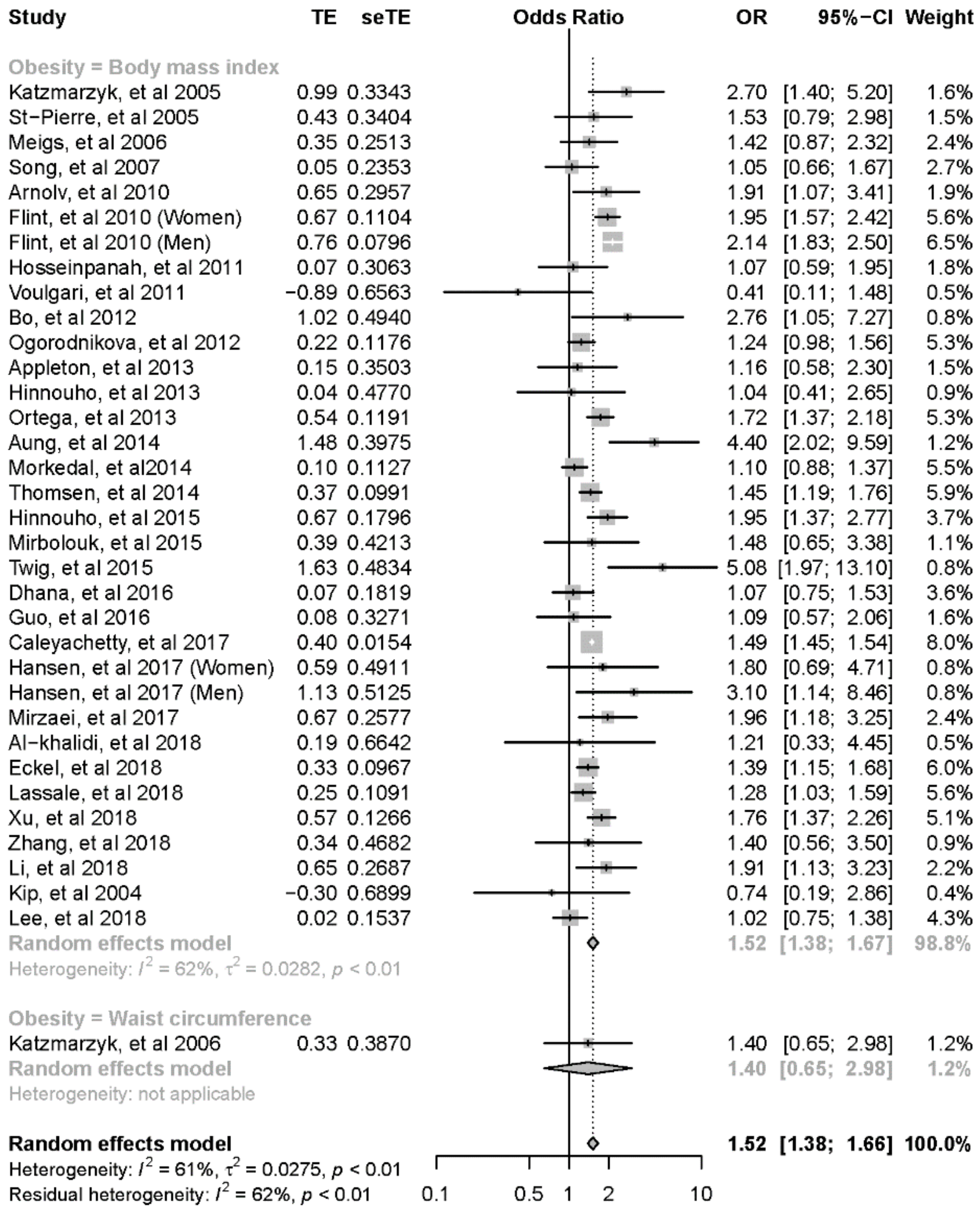

CI, confidence interval; OR, odds ratio; SE, standard error; TE, treatment effect

Figure S3. A Forest plot of cardiovascular disease, comparing participants with metabolically healthy obesity and participants with metabolically healthy normal weight, with a subgroup analysis by different cardiovascular outcomes.

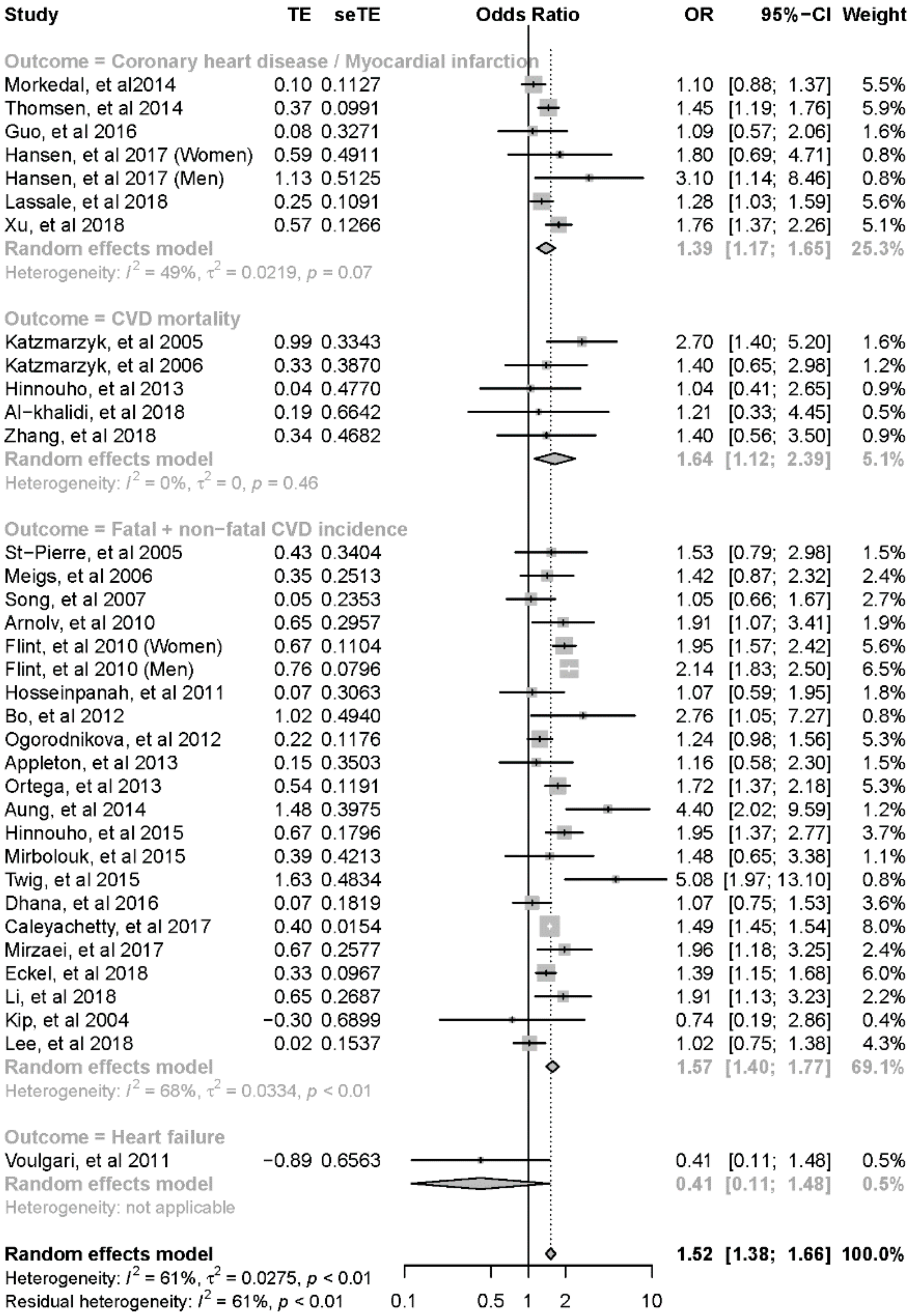

CI, confidence interval; CVD, cardiovascular disease; OR, odds ratio; SE, standard error; TE, treatment effect

Figure S4. Meta-regression bubble plot of the correlation between log odds ratio of cardiovascular disease and age.

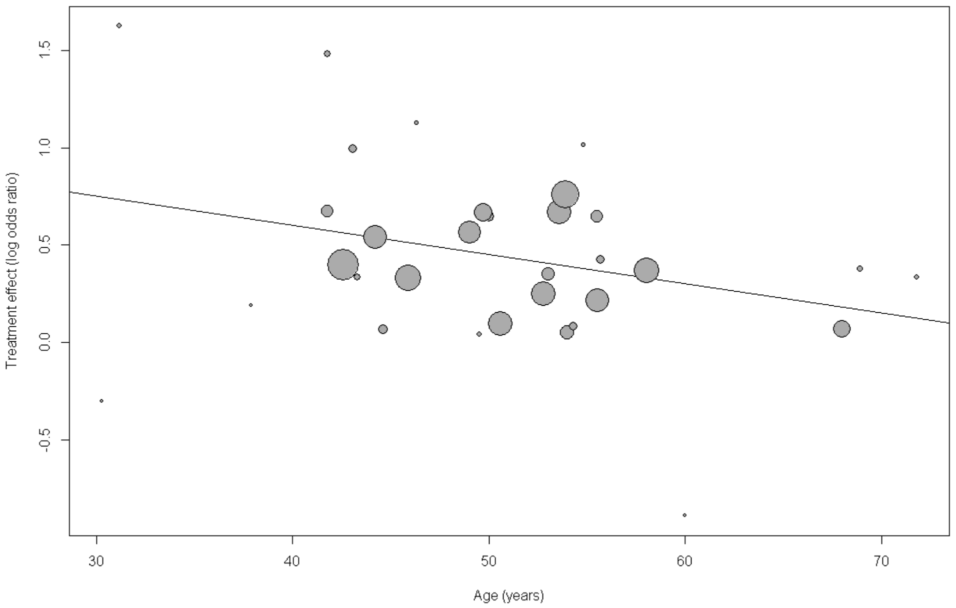

Each bubble represented a study and bubble size represented the sample size of the study. The regression line showed a borderline significant trend of declining risk with age increased.
OR=0.99(0.97; 1.00), *p*=0.06, R^2 (%)=1.6%

Figure S5. Meta-regression bubble plot of the correlation between log odds ratio of cardiovascular disease and smoke.

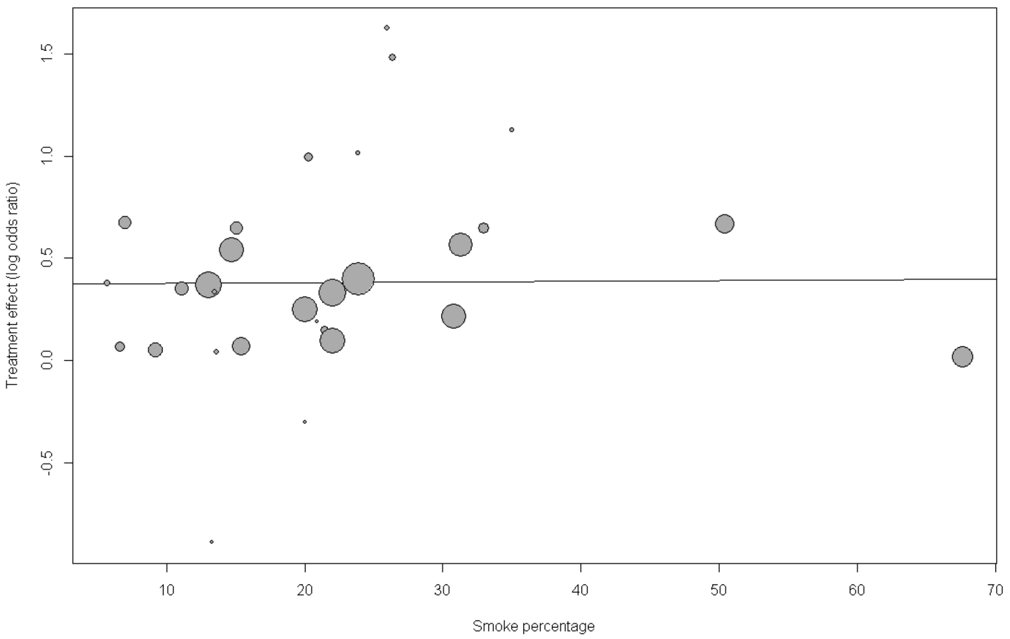

Each bubble represented a study and bubble size represented the sample size of the study. The regression line showed a non-significant trend of increased risk with smoke proportion increased. OR =1.00(0.99; 1.01), p=0.94, R^2 (%)=0%

Figure S6. Meta-regression bubble plot of the correlation between log odds ratio of cardiovascular disease and follow-up duration.

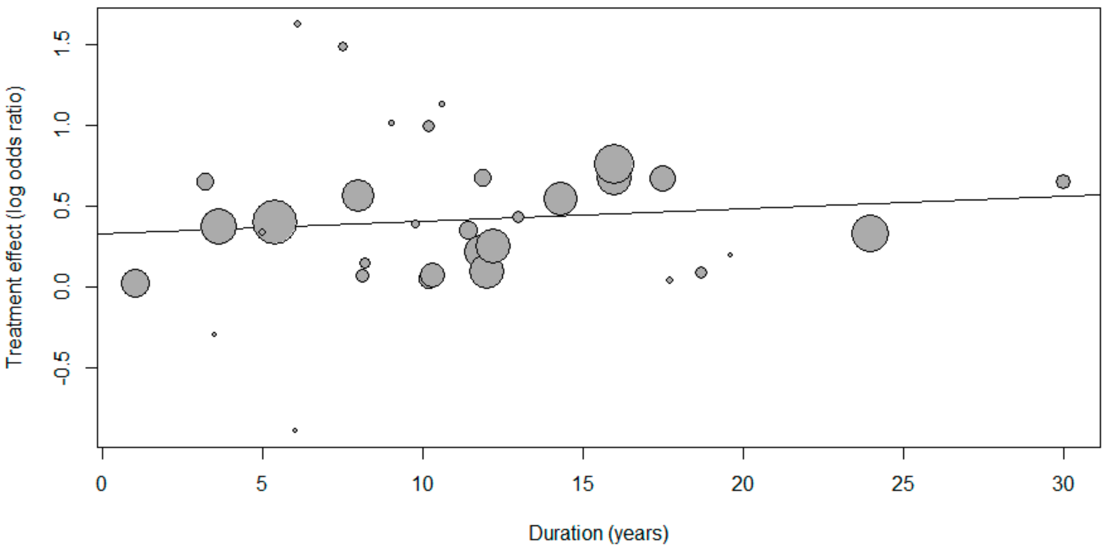

Each bubble represented a study and bubble size represented the sample size of the study. The regression line showed a non-significant trend of increased risk with follow-up year increased. OR =1.01(0.99; 1.03), p=0.43, R^2 (%)=2.2%

Figure S7. The funnel plot standard error and odds ratio of cardiovascular disease showing study dispersion.

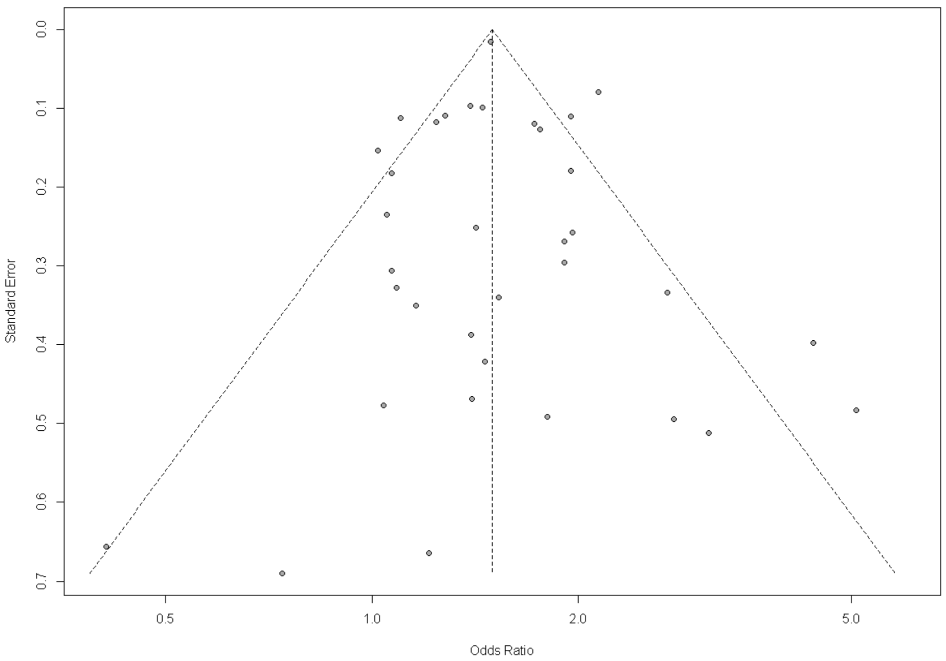

Egger test, slope=0.40, p = 0.73

Table S3. Sensitivity analyses of people with metabolically healthy obesity and risk of cardiovascular disease and all-cause mortality.

	**Risk of cardiovascular disease**			**All-cause mortality**		
	OR (95% CI)	I^2 (%)	Numbers of studies	OR (95% CI)	I^2(%)	Numbers of studies
**Overall**	**1.52(1.38; 1.66)**	61	35	**1.23(1.05; 1.43)**	62	20
**Articles with Newcastle-Ottawa Scale equal or high than 7 points**	**1.54 (1.40; 1.69)**	61	32	**1.23(1.04; 1.44)**	66	17
**CVD without CV mortality**	**1.51 (1.37; 1.66)**	65	30			

Figure S8. Sensitivity analyses of metabolically healthy obesity and risk of cardiovascular disease by omitting each study.

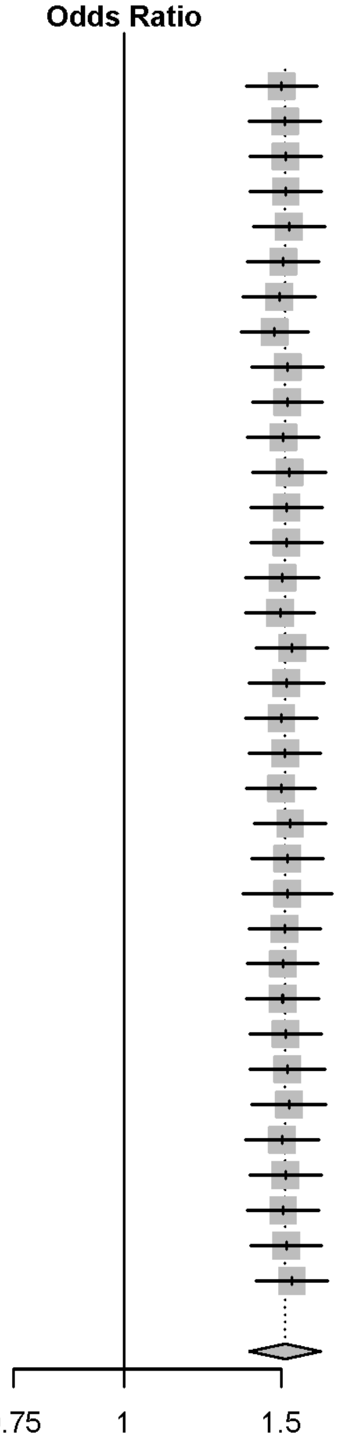

Study	OR	95%−CI
Omitting Katzmarzyk, et al 2005	1.50	[1.37; 1.65]
Omitting St−Pierre, et al 2005	1.52	[1.38; 1.66]
Omitting Katzmarzyk, et al 2006	1.52	[1.38; 1.67]
Omitting Meigs, et al 2006	1.52	[1.38; 1.67]
Omitting Song, et al 2007	1.53	[1.40; 1.68]
Omitting Arnolv, et al 2010	1.51	[1.37; 1.66]
Omitting Flint, et al 2010 (Women)	1.49	[1.36; 1.64]
Omitting Flint, et al 2010 (Men)	1.48	[1.35; 1.61]
Omitting Hosseinpanah, et al 2011	1.53	[1.39; 1.67]
Omitting Voulgari, et al 2011	1.52	[1.39; 1.67]
Omitting Bo, et al 2012	1.51	[1.38; 1.65]
Omitting Ogorodnikova, et al 2012	1.53	[1.39; 1.69]
Omitting Appleton, et al 2013	1.52	[1.39; 1.67]
Omitting Hinnouho, et al 2013	1.52	[1.39; 1.67]
Omitting Ortega, et al 2013	1.51	[1.37; 1.66]
Omitting Aung, et al 2014	1.50	[1.37; 1.64]
Omitting Morkedal, et al2014	1.54	[1.41; 1.69]
Omitting Thomsen, et al 2014	1.52	[1.38; 1.68]
Omitting Hinnouho, et al 2015	1.50	[1.37; 1.65]
Omitting Mirbolouk, et al 2015	1.52	[1.38; 1.66]
Omitting Twig, et al 2015	1.50	[1.37; 1.64]
Omitting Dhana, et al 2016	1.54	[1.40; 1.69]
Omitting Guo, et al 2016	1.52	[1.39; 1.67]
Omitting Caleyachetty, et al 2017	1.52	[1.36; 1.71]
Omitting Hansen, et al 2017 (Women)	1.51	[1.38; 1.66]
Omitting Hansen, et al 2017 (Men)	1.51	[1.37; 1.65]
Omitting Mirzaei, et al 2017	1.51	[1.37; 1.65]
Omitting Al−khalidi, et al 2018	1.52	[1.38; 1.67]
Omitting Eckel, et al 2018	1.52	[1.38; 1.68]
Omitting Lassale, et al 2018	1.53	[1.39; 1.69]
Omitting Xu, et al 2018	1.50	[1.37; 1.66]
Omitting Zhang, et al 2018	1.52	[1.38; 1.67]
Omitting Li, et al 2018	1.51	[1.37; 1.66]
Omitting Kip, et al 2004	1.52	[1.39; 1.67]
Omitting Lee, et al 2018	1.54	[1.41; 1.69]
**Random effects model**	**1.52**	**[1.38; 1.66]**

CI, confidence interval; OR, odds ratio

Figure S9. Cumulative forest plot of risk of all-cause mortality from all included studies.

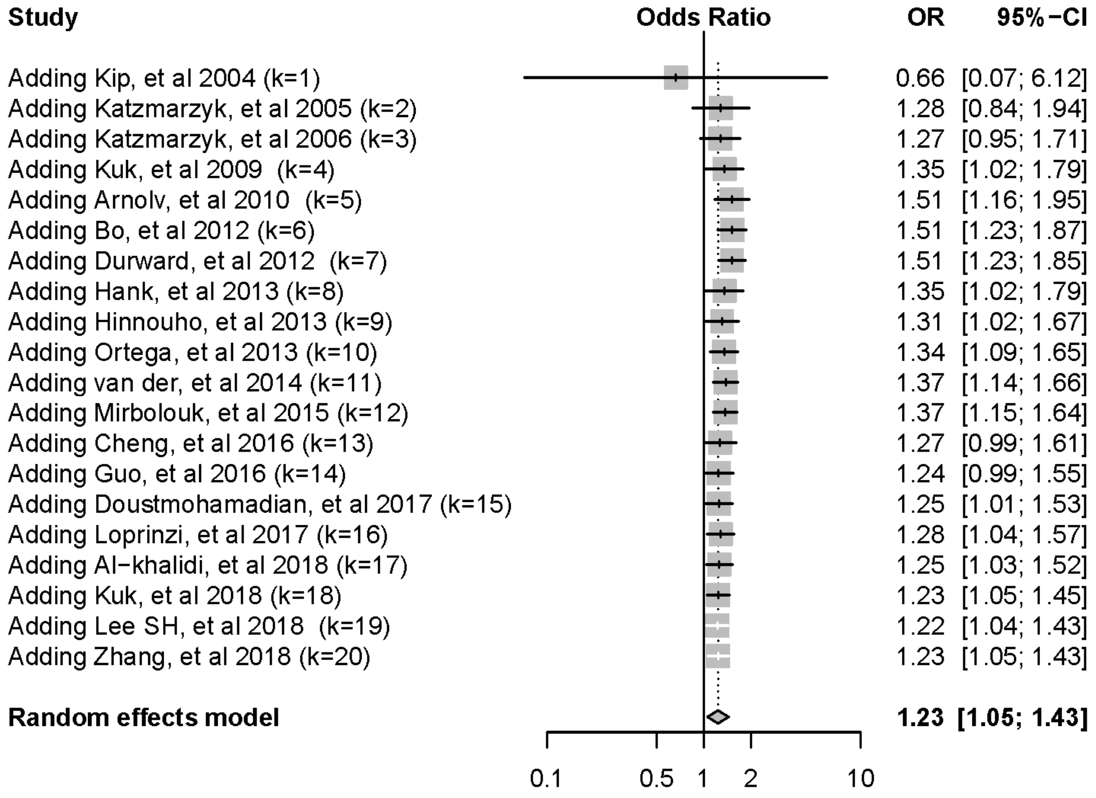

CI, confidence interval; OR, odds ratio

Figure S10. A Forest plot of all-cause mortality, comparing participants with metabolically healthy obesity and participants with metabolically healthy normal weight, with a subgroup analysis by definitions of metabolic health.

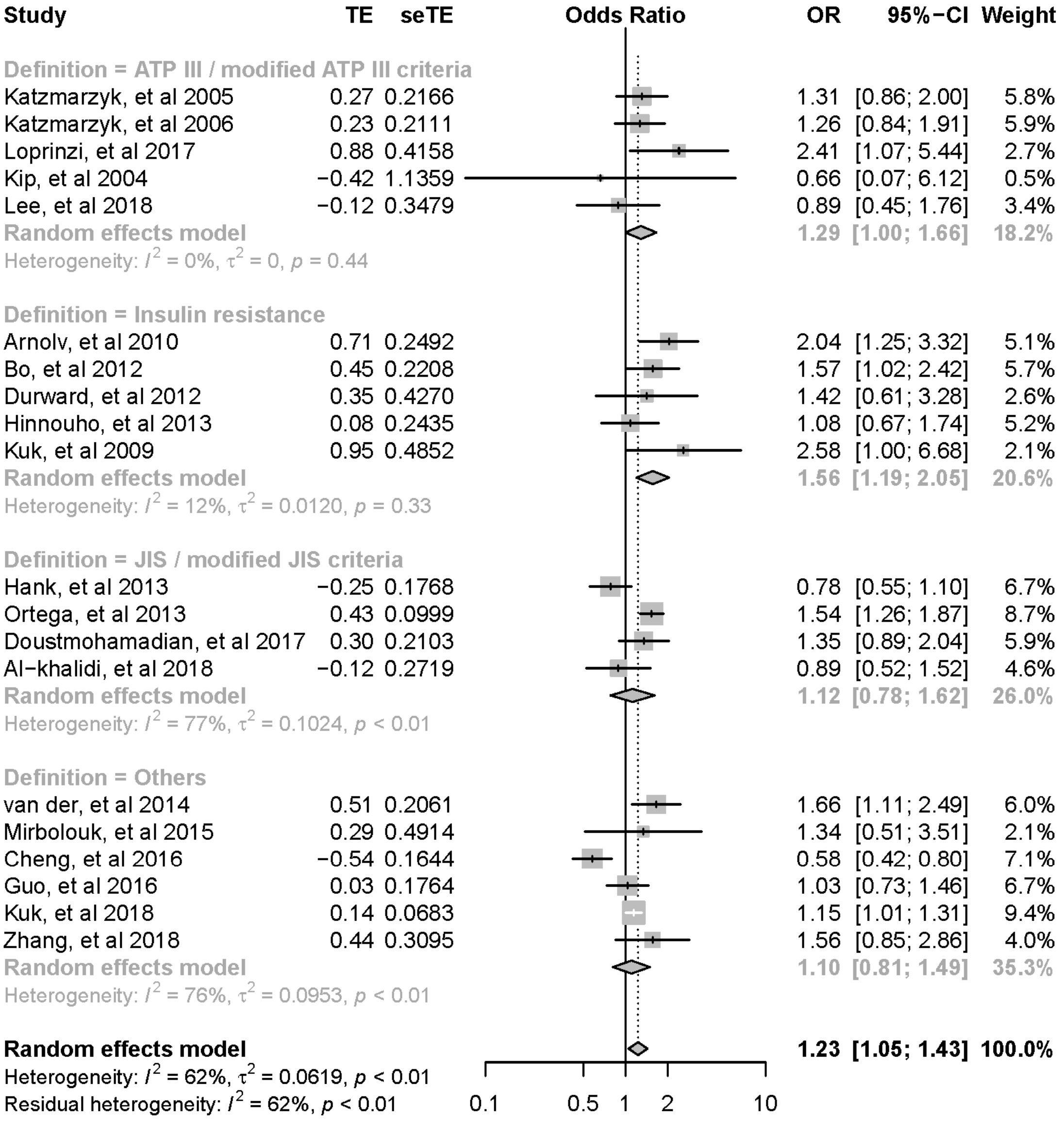

ATP III, Adult Treatment program III; CI, confidence interval; JIS, *Joint Interim Statement;* OR, odds ratio; SE, standard error; TE, treatment effect

Figure S11. A Forest plot of all-cause mortality, comparing participants with metabolically healthy obesity and participants with metabolically healthy normal weight, with a subgroup analysis by definitions of obesity.

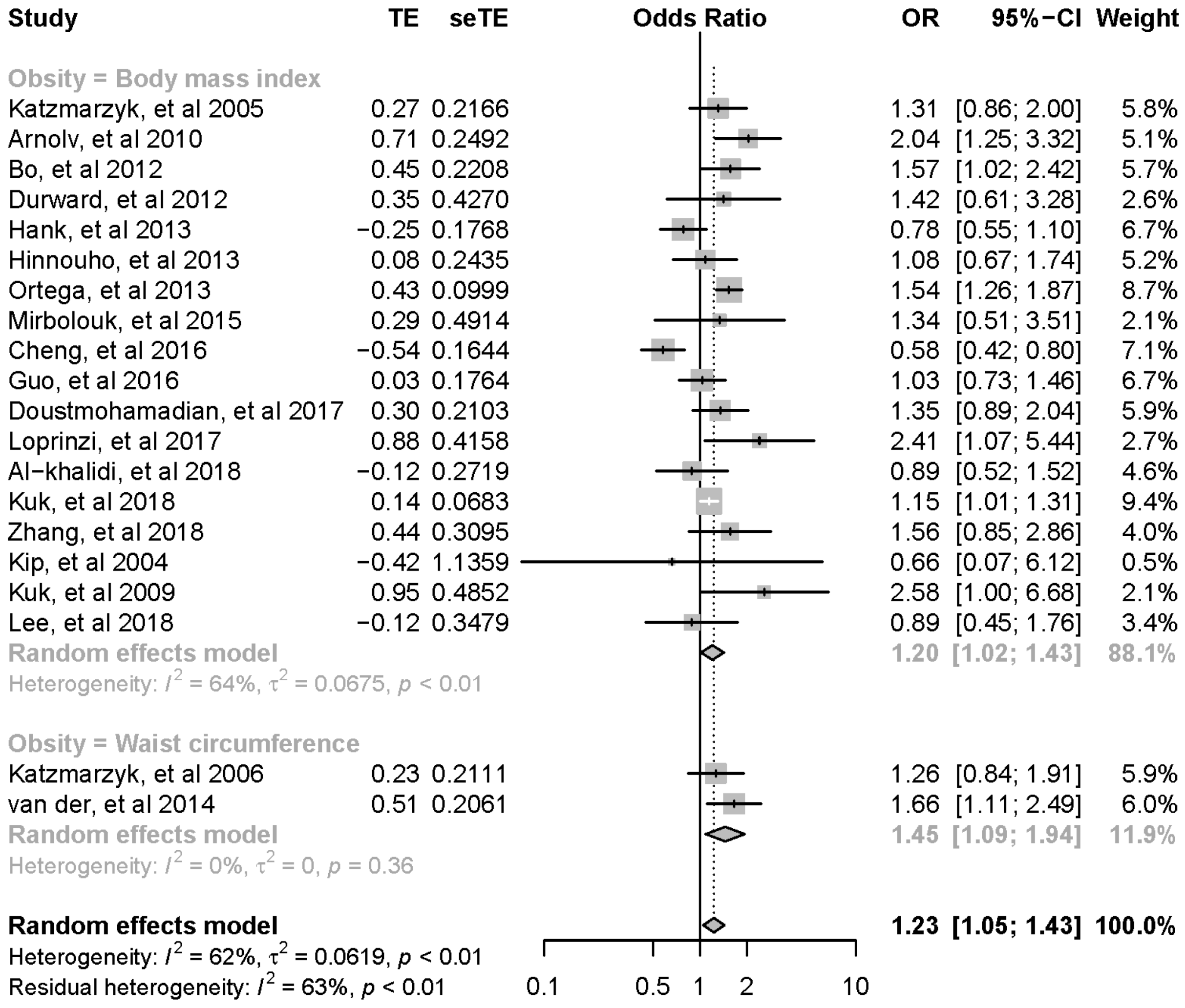

CI, confidence interval; OR, odds ratio; SE, standard error; TE, treatment effect

Figure S12. Dose-responsive analysis of body mass index and the risk of all-cause mortality.

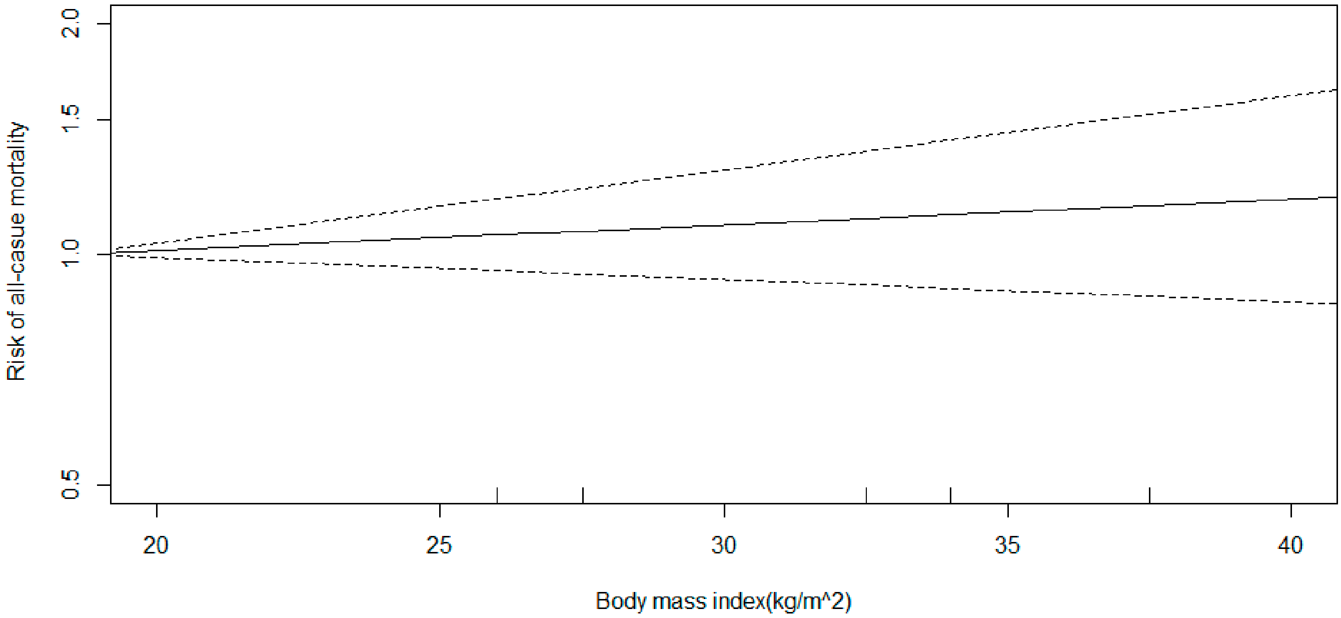

Figure S13. Meta-regression bubble plot of the correlation between log odds ratio of all-cause mortality and age.

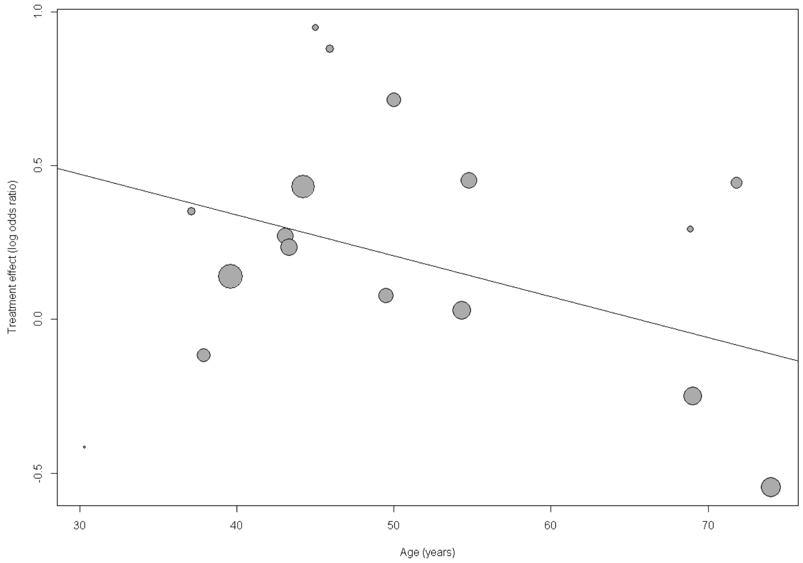

Each bubble represented a study and bubble size represented the sample size of the study. The regression line showed a borderline significant trend of declining risk with age increased.

OR =0.99(0.97; 1.00), *p*=0.07 , R^2 (%)=36.5%

Figure S14. Meta-regression bubble plot of the correlation between log odds ratio of all-cause mortality and smoke.

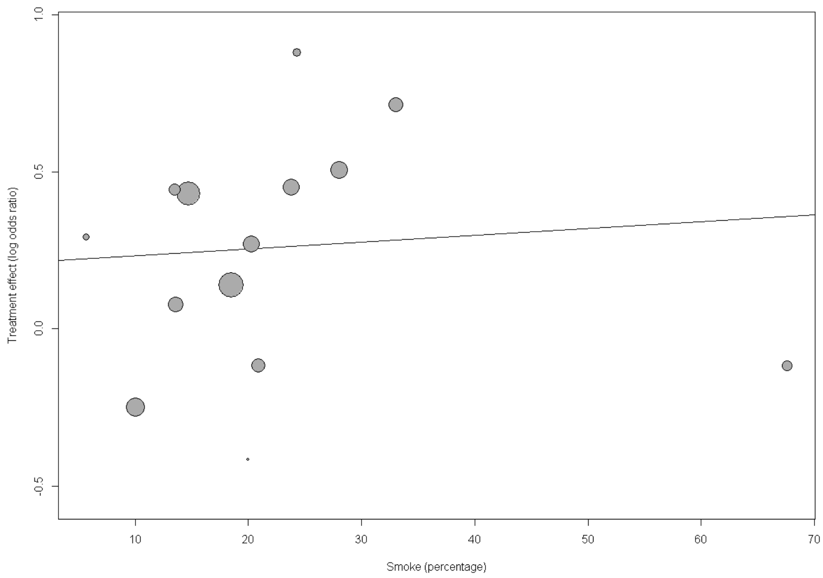

Each bubble represented a study and bubble size represented the sample size of the study. The regression line showed a non-significant trend of increased risk with smoke proportion increased. OR =1.00(0.99; 1.02), p=0.77, R^2 (%)=0%

Figure S15. Meta-regression bubble plot of the correlation between log odds ratio of all-cause mortality and follow-up duration.

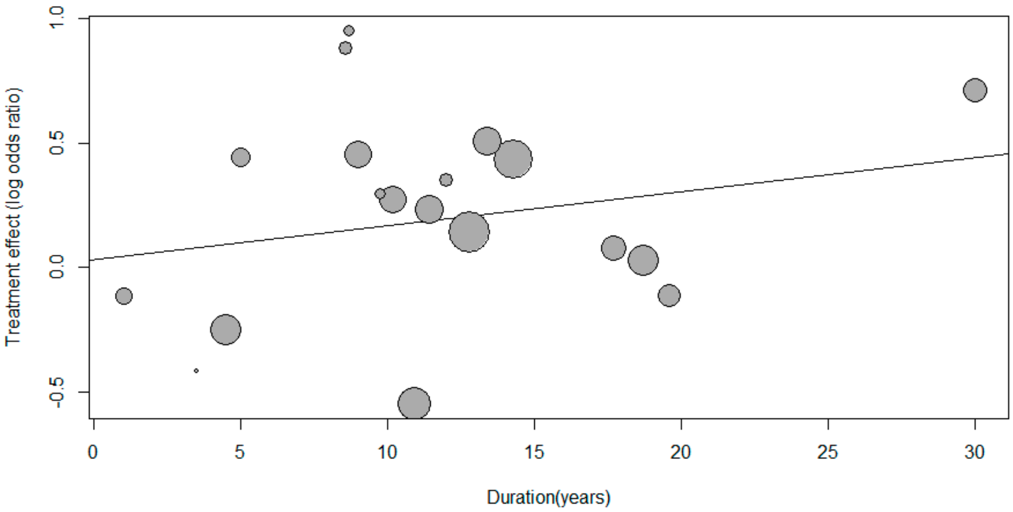

Each bubble represented a study and bubble size represented the sample size of the study. The regression line showed a non-significant trend of increased risk with follow-up year increased. OR =1.01(0.98; 1.04), p=0.33, R^2 (%)=3.7%

Table S4. Meta-Regression analysis of association between covariates and risk of all-cause mortality.

	All-cause mortality							
	Univariate				Multivariate			
	Unadjusted OR (95% CI)	*p* value	I^2 (%)	R^2 (%)	Adjusted OR (95% CI)	*p* value	I^2 (%)	R^2 (%)
**Sex**								
Men	1(Reference)	NA			1(Reference)	NA		
Women	0.50(0.26; 0.98)	0.04	53.1	44.4	0.49(0.28; 0.87)	0.02	53.1	44.4
**Age**, per year increase	0.99(0.97; 1.00)	0.07	57.7	36.5	0.98(0.97; 1.00)	0.02	8.9 [a]	95.5 [a]
**Smoke** status								
Non-smoker	1(Reference)	NA						
Smoker	1.00(0.99; 1.02)	0.77	57.1	0				
Follow up duration, per year increase	1.01(0.98; 1.04)	0.33	67.8	3.7				

CI, confidence interval; NA, not applicable; OR, odds ratio

[a] covariates women and age

Figure S16. **Meta-regression of log odds ratio for all-cause mortality in people with metabolically healthy obesity relative to women proportion.**

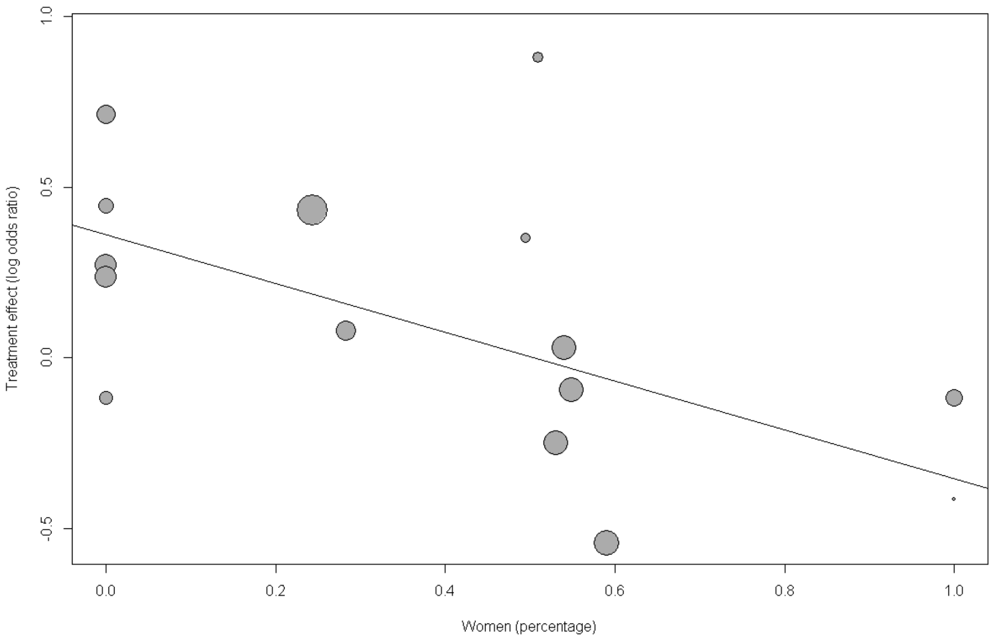

Each bubble represented a study and bubble size represented the sample size of the study. The regression line showed a significant trend of declining risk with larger women proportion.
OR=0.50(0.26; 0.98), ***p*****=0.04** R^2 (%)=44.4%

Figure S17. The funnel plot standard error and odds ratio of all-cause mortality showing study dispersion.

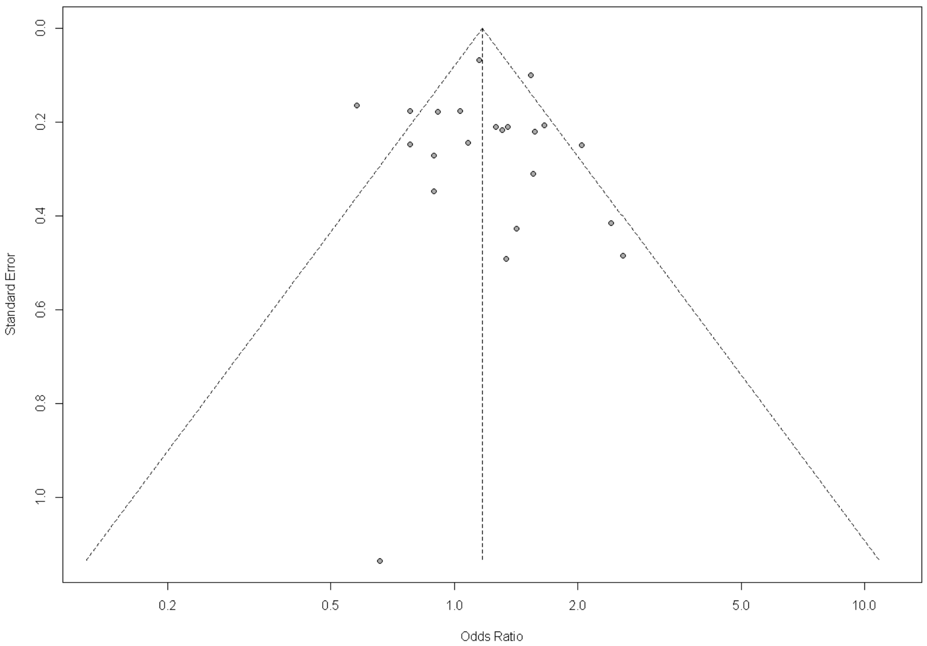

Egger test, slope=0.13, p = 0.62

Figure S18. Sensitivity analyses of metabolically healthy obesity and risk of all-cause mortality by omitting each study.

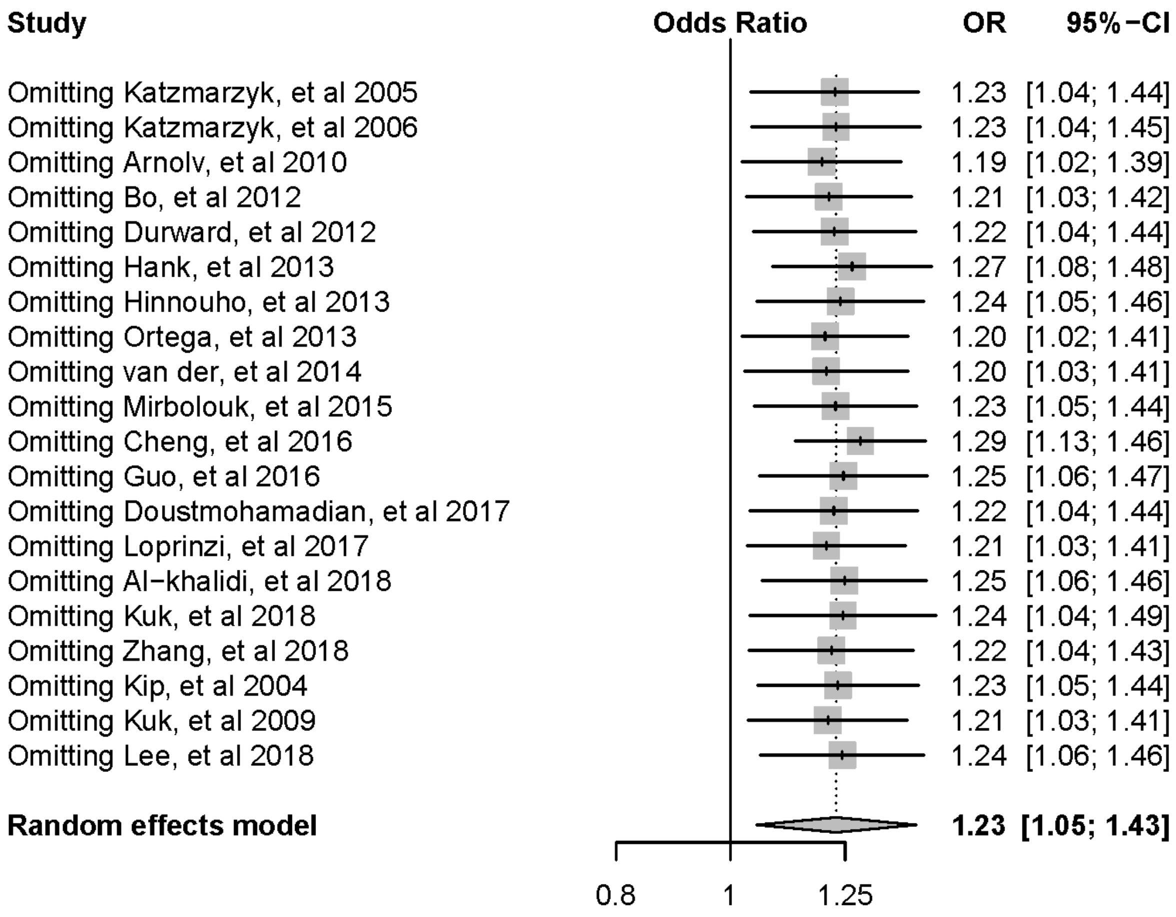

confidence interval; OR, odds ratio
